# Effects of repetitive scanning ultrasound on the intraneuronal dendritic signalling of CA1 pyramidal neurons

**DOI:** 10.3389/fnbeh.2026.1871826

**Published:** 2026-08-07

**Authors:** Bence Pelyvas, Ervin Wolf, Zita Kepes, Ervin Berenyi, Tamas Papp, Attila Somogyi

**Affiliations:** 1 Department of Radiology and Imaging Science, Institute of Medical Imaging, Faculty of Medicine, University of Debrecen, Debrecen, Hungary; 2 Doctoral School of Medical Sciences, University of Debrecen, Debrecen, Hungary; 3 Department of Anatomy, Histology and Embryology, Faculty of Medicine, University of Debrecen, Debrecen, Hungary; 4 Department of Nuclear Medicine and Translational Imaging, Institute of Medical Imaging, Faculty of Medicine, University of Debrecen, Debrecen, Hungary

**Keywords:** CA1 pyramidal cell, computer simulation, hippocampus, neurodevelopment, NEURON simulator, signal attenuation, ultrasound

## Abstract

**Introduction:**

Neural development is regulated by several spatiotemporally changing factors, which are essential for enabling neurons to develop functional networks, during early developmental stages. Additionally, external physical stimuli, like scanning prenatal ultrasound (US) examination, may influence neural development. Aim of our study is to examine potential consequences of diagnostic level ultrasound exposure during the early phase of hippocampal CA1 neural network development by using computational models.

**Methods:**

Using *in silico* modelling, this study explores and compares multiple features of intraneuronal dendritic signal propagation in US-treated and control CA1 pyramidal neurons to look for possible alterations caused by US. We used morphological data of CA1 neurons based on previous morphometric datasets and built high-fidelity subthreshold passive and active segmental cable models of these neurons in the NEURON simulator. To simulate dendritic signalling either a current was injected or a synapse was activated at hundreds of dendritic points of model neurons, eliciting local postsynaptic potentials (PSPs), and multiple descriptors of dendritic impulse propagation between dendritic points and soma were computed.

**Results:**

Our computer models predict that diagnostic level US treatment does not induce changes in specific membrane resistance and capacitance of neuronal membrane. Correlative analyses of simulated dendritic impulse propagation in US treated (UT) and non-treated control neurons (NT) revealed minor differences in attenuations and delays of somatopetally propagating PSPs in the passive model, and these alterations became even less pronounced when hyperpolarization-activated cyclic nucleotide-gated (HCN) cation channels and A-type potassium (K_A_) channels were inserted into the model. Synaptic input pattern recognition between UT and NT neurons showed no significant alterations.

**Discussion:**

We conclude that subthreshold somatopetal signalling properties of US-treated CA1 neuronal membranes remain predominantly at the level of non-treated cells. This conservation is due primarily to HCN channels, contributing to net membrane conductance at rest in an inhomogeneous manner over the somato-dendritic surface. The HCN channels balance the effects of US-induced morphological alterations on dendritic signalling. Conservation of signalling properties align with our independent prediction on the conservation of synaptic integration and input pattern recognition in UT neurons.

## 1 Introduction

Ultrasound (US) is a widely used imaging technique for monitoring foetal development and for screening of congenital malformations during pregnancy. In gynaecological practice, examinations are performed using convex abdominal or transvaginal transducers, operating at frequencies between 3 and 12 MHz. During US imaging, part of the transmitted acoustic energy is absorbed by the tissues, leading to a rise in temperature, which is quantified by the thermal index (TI). In addition, the mechanical effects of US may induce cavitation within tissues; this effect is characterised by the mechanical index (MI). In view of these potential adverse effects, it is imperative that medical foetal examinations are performed with both TI and MI values kept below 1. Moreover, to minimise biomechanical exposure, adherence to the ALARA (As Low As Reasonably Achievable) principle is crucial (Norton, 2016; Maeda, 2014; Quarato et al., 2023).

Previous studies have demonstrated that ultrasonic waves can exert detectable effects on the nervous system at the cellular level. Repeated US exposure using parameters typical of human diagnostic imaging (3 MHz frequency MI and TI values below 1, and a 10-min exposure duration) has been shown to increase c-fos expression, which in turn leads to elevated levels of brain-derived neurotrophic factor (BDNF). BDNF plays a pivotal role in dendritic growth, spine genesis, and the maturation and formation of CA1 pyramidal neurons (Calfa et al., 2012; Papp et al., 2022; Karolyi et al., 2026). Winkler-Ferenczi et al. (2024) further reported an association between US stimulation, comparable to that used in foetal screening, and alterations in dendritic morphology, such as increased dendrite length and spine density (Winkler-Ferenczi et al., 2024). In contrast, Hatch et al. (2016) found no changes in action potential (AP) firing following single or repeated low-frequency US exposure (1 MHz frequency, 0.7 MPa peak rarefactional pressure, 10 Hz pulse repetition frequency, 10% duty cycle and 10 ms pulse length) in CA1 pyramidal cells (Hatch et al., 2016). Furthermore, while repeated US exposure prevents reductions in dendritic structures, it did not significantly influence spine density (Hatch et al., 2016).

In contrast, at US exposure levels exceeding FDA-approved limits, Prieto *et al.* observed that high-frequency US (43 MHz, 50 W/cm^2^) bidirectionally modulated CA1 neuronal firing in a spike-frequency-dependent manner. This observation led to the proposal of the K2P channel hypothesis, which suggests that US activates fast-activating, non-inactivating potassium channels. Subsequent studies have demonstrated that these channels are mechanosensitive potassium channels; consequently, US stimulation induced alterations in the resting membrane potential and action potential properties (Lin et al., 2019; Prieto et al., 2020; Sorum et al., 2021). In their study, Lee *et al.* investigated the effects of an 18.4 MHz US transducer on CA1 pyramidal neurons and reported a correlation between firing rate modulation and pulse repetition frequency (PRF). Their results suggested that the inhibitory effects were primarily driven by the mechanical component of US energy, whereas excitatory effects were predominantly associated with thermal mechanisms (Lee et al., 2025). Wang *et al.* demonstrated that low-frequency US (2.25 MHz) modulates the electrophysiological activity of CA1 pyramidal neurons and local neural circuits, with distinct effects observed under anaesthesia, wakefulness, and locomotion, indicating that US-induced neuromodulation is state-dependent (Wang et al., 2021). Clennell et al. (2021) proposed that low-frequency US (200 kHz) induces a plasticity-like modification in neuronal firing properties. They identified a time-dependent increase in excitability following US stimulation, peaking at 6–8 h and returning to baseline after approximately 12 h, while no substantial alterations in synaptic ultrastructure were observed. Consequently, these findings suggest that US-induced morphological changes may give rise to corresponding alterations in electrophysiological properties.

## 2 Aims

Our aim was to investigate whether the morphological changes observed in scanning US- treated CA1 pyramidal neurons affect dendritic impulse propagation and synaptic input pattern recognition and discrimination.

## 3 Methods

### 3.1 Neuron samples

**Figure 1:**
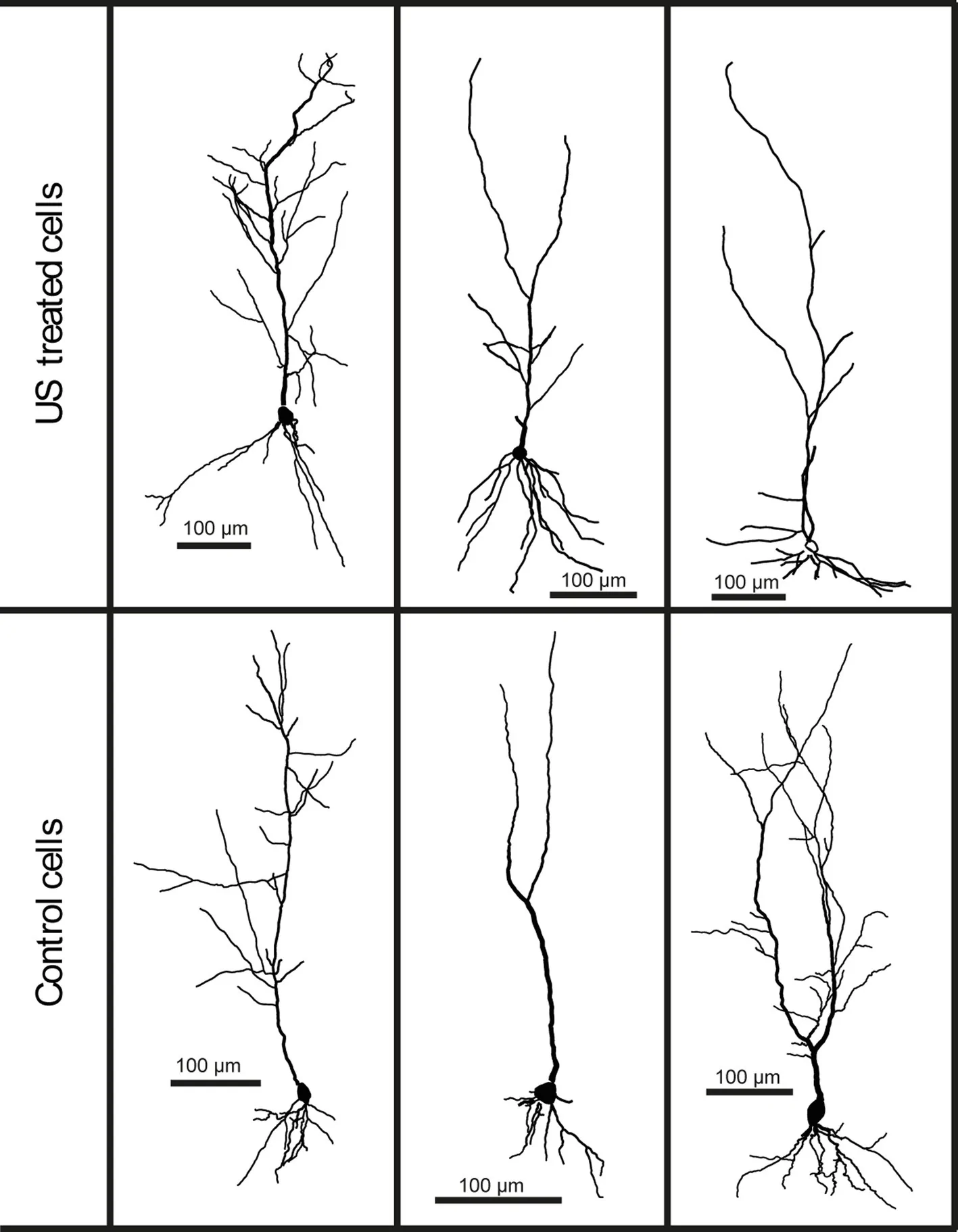
Morphology of hippocampal CA1 pyramidal neurons from US-treated and control mice. Representative neurons were reconstructed with the Neuron Software and are shown with dendritic diameters. Scale bars: 100 μm.

A total of 17 control and 16 US-treated (UT) hippocampal CA1 pyramidal neurons from four 28-day-old UT and three age-matched, untreated (NT) control CD1 (ICR, Charles River, Germany) female mice were used in this study (Figure 1). These neurons were identical to those previously subjected to morphological analysis by Winkler-Ferenczi et al. (2024)Detailed descriptions of in utero electroporation, US treatment (GE Logiq V2, 4C-RS convex transducer (frequency range: 2.0–5.0 MHz, number of elements: 128, convex radius: 60 mmR, FOV: 55°, footprint: 18.3 × 66.2 mm, B-mode imaging frequency: 2.0, 3.0, 4.0, 5.0 MHz, harmonic imaging frequency: 3.0, 4.0, 5.0 MHzFR: 3 MHz, MI: 0.9, T:0.8, duration: 10 min, other parameters: D: 17, DR: 69 dB, AO%: 100, calculated peak negative acoustic pressure was 1.557 MPa, and the average intensity during the exposition was 56 mW/cm^2^), tissue preparation, labelling procedures, and three-dimensional neuronal reconstruction have been reported in the earlier paper.

### 3.2 Compartmentalization of neurons

Based on the traced dendritic morphologies, morphologically faithful, passive, segmental cable models of pyramidal neurons were constructed using the NEURON simulation environment (versions 8.1–8.3) (Hines and Carnevale, 2001, 1997).

Each model neuron consisted of several cylindrical dendritic segments and a single soma compartment with an individually assigned surface area, derived from the reconstruction of the corresponding neuron. The soma surface areas were 218 ± 77 μm^2^ for NT neurons and 140 ± 59 μm^2^ for UT neurons. The number of dendritic segments varied depending on the branching pattern, arborization complexity, and dendritic size. In NT neurons, basal and apical dendritic arbours comprised 5–44 and 2–89 segments, respectively, whereas in UT neurons these ranges were 14–46 (basal) and 16–106 (apical).

### 3.3 Modelling the spines

A reduction in dendritic spine density and number has been observed in CA1 pyramidal neurons (Winkler-Ferenczi et al., 2024), therefore, to account for possible effects of dendritic spines on impulse conduction, spines were incorporated into the computational neuron models. During spine modelling, the specific membrane capacitance and membrane conductance (capacitance and conductance per unit membrane area) of each dendritic compartment were proportionally adjusted to account for the increase in effective dendritic surface area caused by the presence of spines. This approach captures the electrical consequences of dendritic spines without explicitly altering the diameter or length of the cylindrical compartments (Holmes, 1989; Jaslove, 1992; Larkman, 1991; Schmidt-Hieber et al., 2007; Shelton, 1985; Somogyi et al., 2016; Stuart and Spruston, 1998). As the first step of this procedure, the relative increase in surface area *(q)* attributable to dendritic spines was calculated for each dendritic compartment (Equation 1):


$$q=\frac{\left(\mathrm{As}+\mathrm{Ad}\right)}{\mathrm{Ad}}$$ (1)

where *A_s_* denotes the total surface area of dendritic spines associated with a given dendritic compartment, and *A_d_* represents the surface area of the corresponding “smooth” dendritic compartment in the absence of the spines. Subsequently, the specific membrane capacitance (C*_m_) and membrane conductance (G*_m_) of each dendritic compartment were adjusted according to the relationships C*_m_ = C_m_ · q and G*_m_ = G_m_ · q, where *C_m_* and *G_m_* denote the specific membrane capacitance and conductance of the corresponding dendritic compartment in the absence of spines. This approach represents a general method for incorporating the global effects of dendritic spines on impulse propagation within passive cable models. The approach was suitable for the present study, as our aim was to analyse impulse propagation along dendrites rather than within individual spines. In the computational models of UT and NT pyramidal neurons, linear spine densities were set to 1.607 and 1.218 spines/μm in basal dendrites and 1.057 and 1.551 spines/μm in apical dendrites, respectively. Spine surface areas were set to 0.65 and 0.79 μm^2^/spine in basal dendrites; and 0.656 and 1.38 μm^2^/spine in apical for UT and NT neurons, respectively. These parameters were derived from quantitative measurements reported by Winkler-Ferenczi et al. (2024).

### 3.4 Membrane models of neurons

At present, no comparative data are available regarding the effects of prenatal US exposure on the spatial distribution and kinetics of voltage-dependent conductances in the soma-dendritic membrane of CA1 pyramidal neurons. Therefore, dendritic impulse propagation was first examined by using a passive cable model and then we applied standard subthreshold active membrane models.

To set up passive cable models, uniform distributions of leakage conductances were employed. In this type of neuronal membrane models, the membrane resistance per unit area was assumed to be identical across the soma-dendritic membranes (Rms = Rmd). Previous findings indicate that scanning US does not alter the input resistance and membrane time constant of CA1 pyramidal neurons (Hatch et al., 2016). Therefore, to determine the individual specific membrane resistances (R_m_) for each UT and NT neuron model, the membrane resistance was iteratively adjusted until the electrophysiologically measured (Lamsa et al., 2007) mean somatic input resistance (107 ± 16 MΩ) was reproduced in both cell groups. Subsequently, the specific membrane capacitance (*C_m_*) was set to be equal across the entire soma-dendritic surface. For each model neuron, the time constant (τ) was fitted to the electrophysiologically measured value (29.8 ± 6.8 ms) in both UT and NT cells by adjusting *C_m_* in increments of 0.1 μF/cm^2^. The specific axial resistance of the cytoplasm *(R_a_)* was set to 150 Ωcm in all simulations (Trevelyan and Jack, 2002; Kabaso et al., 2009).

To construct subthreshold active cable models of ultrasound-treated (UT) and non-treated (NT) neurons, we incorporated hyperpolarization-activated cyclic nucleotide-gated (HCN) and A-type potassium (K_A_) channels alongside standard leakage conductances. These primary active conductances operate below the action potential threshold and dynamically interact with ultrasound-induced geometric variations in a location-dependent manner. Accordingly, we implemented established canonical models of HCN and K_A_ channel kinetics and spatial distributions. To strictly constrain the parameter space and isolate the core mechanisms governing subthreshold dendritic integration, we deliberately omitted suprathreshold voltage-gated ion channels and secondary subthreshold currents.

HCN channels were uniformly distributed across the soma and basal dendrites with a base conductance of 1.5 × 10^−5^ S/cm^2^. Within the apical arbour, HCN density increased linearly as a function of distance from the soma (d, in μm), scaled by the function 1 + 3d/100 (Magee, 1998). A-type potassium currents were modeled using distinct proximal (kap) and distal (kad) mechanisms to account for established distance-dependent kinetic shifts along the somatodendritic axis (Hoffman et al., 1997). A uniform proximal conductance of 0.03 S/cm^2^ was applied to the soma and basal dendrites. In the apical dendrites, K_A_ density increased linearly according to the function 0.03 × (1 + d/100) S/cm^2^ to reflect known physiological gradients. Proximal kinetics were restricted to apical compartments within 100 μm of the soma, while distal kinetics governed the macroscopic current (*I_A_*) at distances ≥100 μm, consistent with foundational computational implementations (Migliore et al., 1999; Migliore et al., 2005). Finally, all regional active conductances, leakage conductances, and specific membrane capacitances were multiplied by a local *q*-factor to account for the relative surface area increases contributed by dendritic spines. The resting membrane potential was initialized and maintained at −65 mV. To fit these active models to our experimental baseline impedance and time constant values, we fitted specific leakage conductances and capacitances using 10 pA hyperpolarizing current steps, mirroring the protocol used for our purely passive models. Furthermore, to achieve a biologically realistic range of leakage resistances and prevent the mathematical short-circuiting of the apical dendrites, we scaled the maximal HCN channel conductances as recently recommended for mouse models (Vitale et al., 2023). Despite these adjustments, one NT and two UT model neurons could not be successfully fitted to the population-mean electrophysiological targets. Rather than a limitation of the computational framework, this outcome reflects the extreme natural morphological variance inherent to biological tissue. In these specific cells, the combination of exceptionally large somata and a high density of distal apical HCN channels created a massive active shunt, rendering the membrane too leaky to achieve the targeted mean somatic input resistance without mathematically forcing specific membrane parameters into non-physiological ranges. To accommodate this natural variance while maintaining strict biological plausibility, we relaxed the fitting targets for these three extreme cases to fall within *μ* ± 1σ, a threshold that encompasses the central ~68% of expected biological variance of the population mean. Under these broadened physiological constraints, two of the variant neurons were successfully fitted. Only a single UT neuron, which possessed the largest reconstructed somatic surface area in the dataset, remained impossible to fit within a physiological range of membrane resistances. Consequently, this single neuron was excluded from further downstream analysis as a structural outlier.

### 3.5 Initiation of postsynaptic potentials (PSPs) and measures of dendritic signal transfer

*In the passive model* postsynaptic potentials (PSPs) were modelled by steady-state and 50 Hz sinusoidal current injections applied at dendritic locations. The distance between neighbouring injection sites, representing synaptic loci, was kept below 14 μm or 0.2 space constants, resulting in 9–446 injection sites per neuron, depending on dendritic size and arborization pattern. This approach enabled a systematic assessment of dendritic signal transmission. Somatopetal subthreshold dendritic impulse propagation, was analysed and compared between UT and NT pyramidal neurons by examining current transfer, voltage transfer, and delays of PSPs. Voltage transfer was defined as the ratio of somatic to dendritic PSP amplitude at the site of current injection. Current transfer was defined as the ratio of the electrical charge reaching the soma to the total charge injected at the dendritic site. Total delay (delay in the followings) was defined as the time required for synaptic current to elicit a PSP and to propagate from the dendritic site to the soma and was composed of two components: (i) local delay, corresponding to the time required for the injected current to generate a local dendritic PSP; and (ii) propagation delay, corresponding to the time required for the PSP to travel from the dendritic site to the soma. *Local delay* was measured as the time difference between the centroids of the voltage-time and current-time curves at the injection site, whereas *propagation delay* was calculated as the time difference between the centroids of the voltage-time curves recorded at the soma and at the dendritic injection site (Agmon-Snir and Segev, 1993; Zador et al., 1995). Transfers and delays were analysed separately for apical and basal dendrites to account for the different morphological alterations in basal and apical dendrites observed in UT animals (Winkler-Ferenczi et al., 2024). To compare dendritic signal transmission between UT and NT neurons, two types of comparison graphs were constructed: (1) First, to assess the dependence of transfer and delay measures on dendritic location, transfers and delays were plotted as functions of the path distance between the soma and the PSP generation site. (2) Second, to evaluate the relative contribution of specific transfer and delay values across the dendritic tree, distributions of dendritic surface area, serving as a proxy for synaptic distribution, were plotted as functions of transfer and delay values. Finally, to determine whether the main findings were independent of within-group variability in neuronal size, distance-dependent attenuation and delay measures, as well as dendritic surface area distributions as functions of transfer and delay were also analysed using normalized scales. In these analyses, path distances and transfer/delay values were expressed as percentages of their respective maximum values within individual neurons.

*In the active subthreshold models* calculation of steady-state voltage and current transfers and sinusoid voltage transfers was based on the same definitions described earlier. In case of delays, dendritic PSPs were initiated by an alpha synapse (maximum conductance change 1 nS, 2 ms time to peak and 0 mV reversal potential) mimicking glutamatergic AMPA synapses to increase realism of simulations even further (Magee and Cook, 2000; Spruston et al., 1995). Following activation of this synapse, propagation delay was calculated as the time difference between the simulated peak somatic and dendritic potentials.

### 3.6 Quantitative analysis of synaptic input pattern recognition in ultrasound treated and untreated neurons

Synaptic input pattern recognition abilities of neurons have previously been quantitatively analysed using computational modelling by de Sousa et al. (2015). In that study, a large population of model neurons with diverse morphologies and either passive or active membrane properties was examined using a Hebbian learning framework based on use-dependent synaptic facilitation. During training periods, randomly selected subsets of synapses were repeatedly activated, leading to a gradual increase in their synaptic conductance. Following training, neuronal output was evaluated by comparing somatic excitatory postsynaptic potential (EPSP) amplitudes or action potential counts evoked by trained input patterns versus novel inputs. Across both passive and active membrane models, synaptic input pattern recognition capacity was found to be inversely related to two key metrics: the mean electrotonic distance of synapses from the soma and the within-cell variance of these distances (de Sousa et al., 2015). In the present study, the mean and variance of synaptic electrotonic distances were calculated for both UT and NT neurons based on the distances of dendritic current injection sites (simulated synaptic loci) from the soma in passive models of each reconstructed neuron.

### 3.7 Ethics approval

The animal experiments conducted as part of our previous study were reviewed and approved by the Animal Experimentation Committee of the University of Debrecen. The study was conducted in accordance with the ARRIVE guidelines. This study utilised in silico modelling with data from the preceding study, thus obviating the necessity for ethical approval.

### 3.8 Statistical analysis

Statistical analyses and figure generation were performed using Microsoft Office (Microsoft corp.) and PAST software (Hammer et al., 2009). For pairwise comparisons of means and variances of electrotonic distances, as well as specific membrane resistances and capacitances of UT and NT neurons the Mann–Whitney test was applied. In comparison graphs depicting the distance-dependence of transfers or delays of somatopetally propagating PSPs and dendritic surface areas associated with identical transfers or delays, two-way ANOVA test was used to identify overall alterations in dendritic signalling in UT neurons. Bonferroni *post hoc* tests were subsequently used to determine specific path distance regions or transfer/delay intervals in which UT and NT neurons differed significantly. *Post hoc* analyses were restricted to intervals containing at least three data points from both neuronal populations. Distributions of dendritic length and surface area as a function of path distance measured from the soma were compared by Kolmogorov–Smirnov tests.

In all analyses, the threshold for statistical significance was set at *p* < 0.05. Quantitative data are presented as arithmetic means ± standard error of the mean (SEM), with SEM values indicated by error bars in the figures. The proportion of path distance regions and transfer/delay intervals exhibiting statistically significant differences between UT and NT neurons was used as a quantitative measure of the degree of alterations in dendritic signalling.

## 4 Results

### 4.1 Dendritic surface area reflects synapse distribution in CA1 pyramidal neurons

Previous work by Winkler-Ferenczi et al. (2024) demonstrated that scanning US alters the dendritic morphology of CA1 pyramidal neurons. Here, we sought to explore the functional consequences of these morphological changes by quantitatively analysing somatopetal dendritic signal transmission. To this end, descriptors of dendritic signalling–specifically transfers and delays of PSPs-were analysed as functions of the path distance of synaptic input site from the soma. In addition to assessing the location dependence of these descriptors, we aimed to weight them according to the relative abundance of synapses exhibiting similar signalling properties toward the soma. For this purpose, the fraction of synapses was approximated by the fraction of dendritic surface area from where PSPs with comparable descriptors originated. This approach is supported by the morphological organisation of CA1 pyramidal neurons, in which more than 95% of excitatory synapses are located on dendritic spines (Bloss et al., 2018). Moreover, approximately 90% of spines receive a single synapse, whereas spines lacking synaptic contacts are rare (<2%) (Schikorski and Stevens, 1999). Together, these observations suggest that the spatial distribution of dendritic spines provide a reliable proxy for the distribution of excitatory synapses along the dendritic tree.

**Figure 2:**
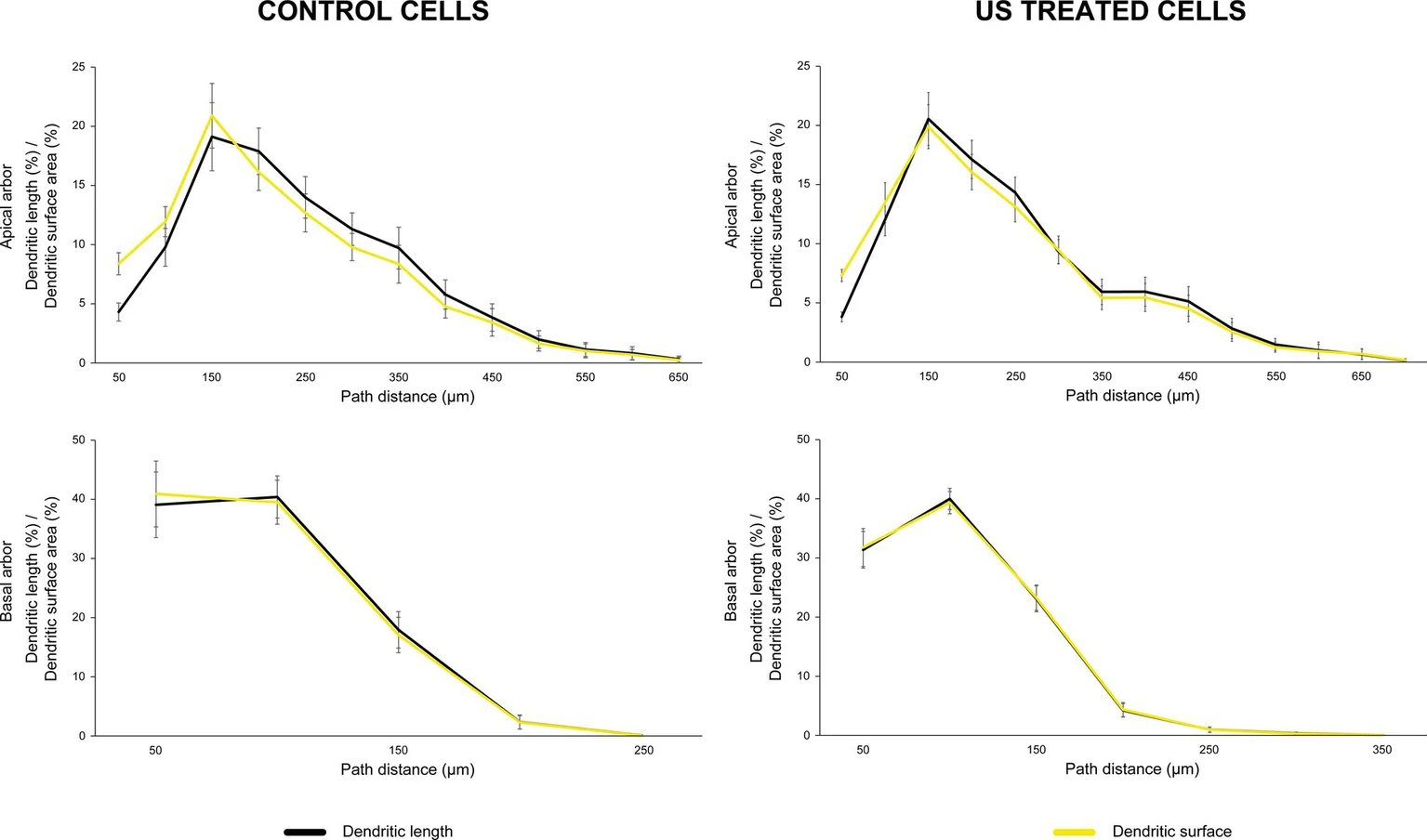
Distributions of dendritic length and surface area as a function of path distance measured from the soma. Distributions of dendritic length (black) and surface area (yellow) plotted against path distance from the soma. The two distributions are statistically indistinguishable (Kolmogorov–Smirnov test, *p* > 0.91).

Consistent with this notion, linear spine density was previously shown to be nearly constant along dendrites in both NT and UT neurons (Winkler-Ferenczi et al., 2024), suggesting that dendritic length distributions closely approximate spine and excitatory synapse distributions as well. We therefore compared the cumulative distributions of dendritic length and dendritic surface area as functions of path distance from the soma in apical and basal dendrites of 17 control and 16 US treated pyramidal neurons. No significant differences were detected between these distributions in either group (Figure 2, Kolmogorov–Smirnov test, *p* > 0.91). These results indicate that the proportion of total dendritic surface within a given path distance range can be used as a valid estimate of the proportion of excitatory synapses received by that dendritic region in both control and ultrasound-treated CA1 pyramidal neurons.

### 4.2 Simulation of membrane properties

**Figure 3:**
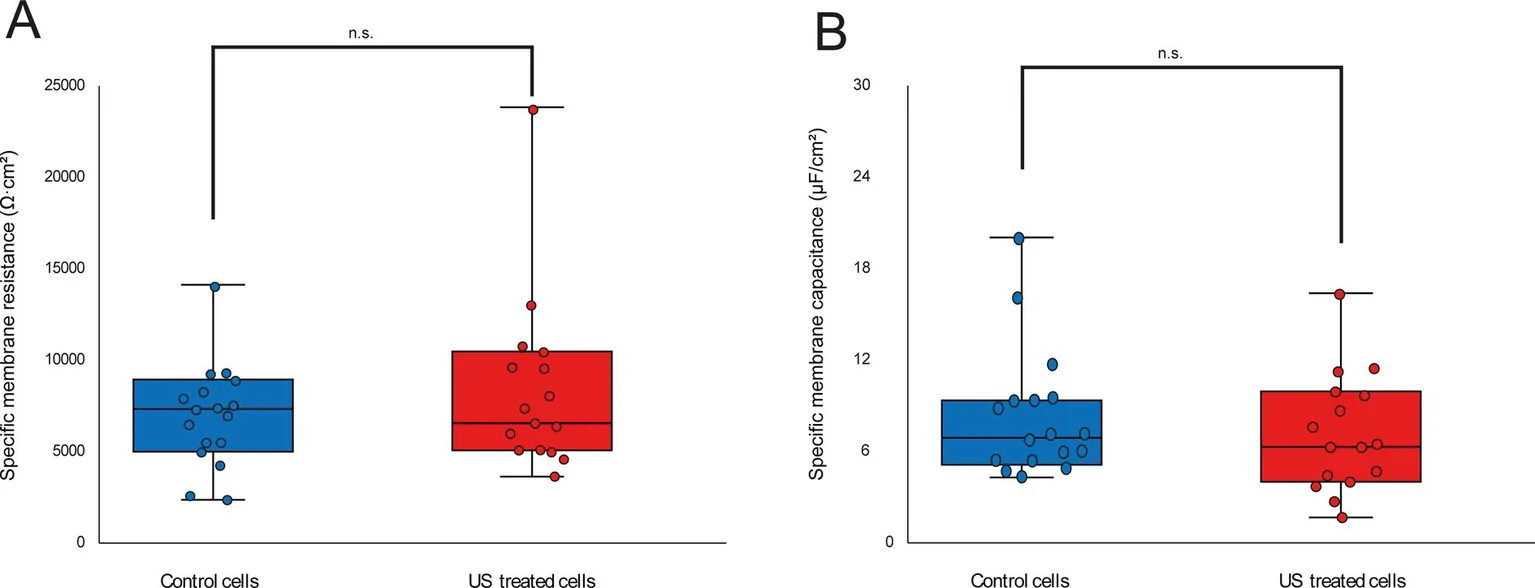
Specific membrane resistance and capacitances in NT and UT CA1 pyramidal neurons based on fittings in passive models. Dot-and-box plots comparing specific membrane resistance **(A)** and capacitance **(B)** in NT (blue) and UT (red) neurons. No significant differences were found between groups for either parameter (Mann–Whitney test, *p* = 0.55 for both resistances and capacitance). Lower and upper boundaries of the boxes indicate the 25th–75th percentiles; the central line indicates the median, and whiskers denote the maximum and minimum values.

To construct segmental cable models of NT and UT neurons, we estimated the specific leakage resistance and capacitance, parameters that cannot be directly obtained from conventional intracellular electrophysiological recordings. These values are inferred using anatomically detailed compartmental models implemented in NEURON simulation environment, by fitting experimentally measured somatic input resistances and membrane time constants (see Methods). Mann–Whitney test did not detect any significant differences between NT and UT neurons in either specific membrane resistance (*p* = 0.55) or specific membrane capacitance (*p* = 0.55) (Figure 3).

### 4.3 Analysis of dendritic signalling in CA1 pyramidal neurons

Given the pronounced morphological changes previously reported in CA1 pyramidal neurons following US treatment, most prominently affecting basal dendrites (Winkler-Ferenczi et al., 2024) and the absence of changes in passive membrane properties, we next examined how these structural alterations influence intraneuronal dendritic signal propagation. For quantitative and comparative analysis two-types of comparison graphs were used. One type displays the location dependence of the descriptors of generated PSPs, while the other type shows the proportion of dendritic synapses (~approximated by the distribution of dendritic surface area) with similar descriptors of signalling. The rationale behind this method is that either the location dependence of signalling properties or the proportion of synapses with similar PSP transfers or delays is significantly altered in UT neurons, such changes can be attributed to the effects of scanning US. Steady-state- and sinusoidal voltage transfers, current transfers and delay were used as descriptors of propagation of locally generated PSPs. The ability of dendritic synapses to affect membrane potential dynamics at the soma (and at the adjacent axon hillock) can be measured by voltage transfers. If all other variables are similar, synapses at loci with higher values of voltage transfer may have a greater impact on somatic potential and consequently firing activity of postsynaptic neurons than synapses at loci with smaller values of voltage transfer.

Steady-state voltage transfer measures attenuation of PSPs with relatively long time course (e.g., mediated by N-methyl-D-aspartate (NMDA) receptors). However, voltage transfer depends on the frequency of the signal, because membrane capacitance is not zero. Therefore, PSPs with short time constants (e.g., elicited at synapses mediated by AMPA receptors) attenuate differently. To account for this frequency-dependence of signal propagation we also computed 50 Hz sinusoidal voltage transfers.

When voltage perturbations are small, the amount of electrical charge reaching the soma predicts the chances of an action potential generation better than the amplitude of the somatic membrane potentials (Jack et al., 1975). To account for this, current transfer was measured as a fraction of electrical charge reaching the soma relative to the total charge injected locally at the synaptic site. Another interpretation is based on equality between the somatopetal charge transfer from a dendritic point to the soma and the somatofugal voltage transfer from the soma to the same dendritic point (Zador et al., 1995). Thus, based on this equality, current transfer can also be interpreted as the efficiency of passive back propagation of voltage changes along the dendrites.

Any alteration in the delay of PSPs can also affect the summation of dendritic signals arriving at the soma. Summation of simultaneous synaptic inputs arriving at the soma may be different if delays of PSPs get altered as these changes may prevent PSPs from effective summation (and subsequent action potential generation) because they may arrive at the soma in less-overlapping time frames.

As the extent of morphological alterations in the apical and basal dendritic arbours are different, the apical and basal dendrites were examined separately. We investigated dendritic signalling by plotting eight comparison graphs for each transfer property (current transfer, steady-state and sinusoidal voltage transfer) and for the delay of PSPs.

In the following, our analysis of dendritic signalling is divided into three parts. First, we consider the effects of US-induced morphological alterations by simulating entirely passive uniform neuronal membranes, without the confounding effects of any voltage-dependent conductances. Next, we compare UT and NT neurons with HCN and K_A_ voltage-dependent channels in their membranes. Any alteration in signalling observed between UT and NT model neurons in this scenario, is the effect of US-induced morphological variations enhanced or limited by the two major subthreshold active ion channels. Finally, we isolate the effects of HCN and K_A_ channels on subthreshold dendritic impulse propagation by comparing model neurons with UT morphology with and without HCN and K_A_ channels.

### 4.4 Effects of US-induced morphological alterations on transfers of PSPs in passive models

**Figure 4:**
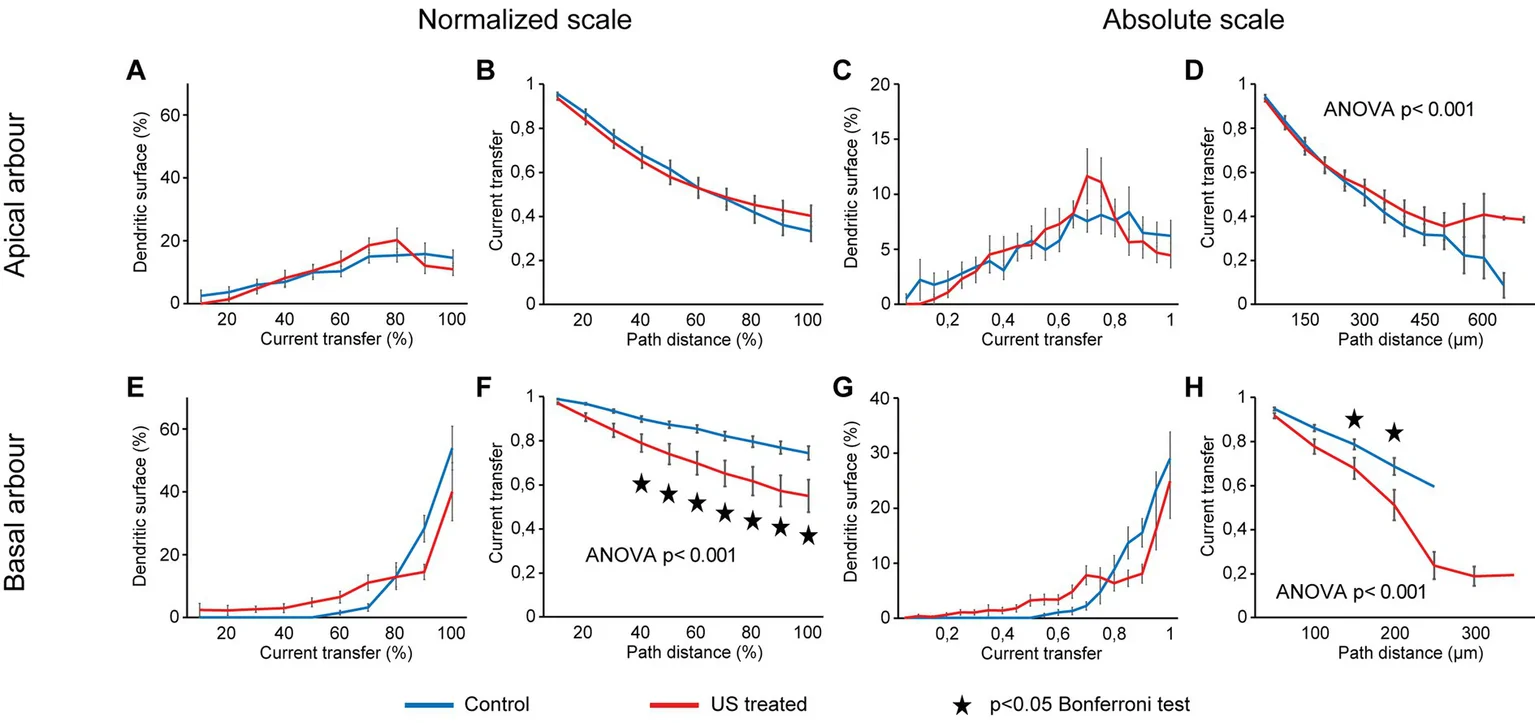
Altered current transfer of locally generated PSPs in passive models of UT CA1 pyramidal neurons. Comparison graphs illustrate alterations in dendritic impulse propagation of UT (red) relative to control NT (blue) CA1 pyramidal neurons. Two types of plots are shown: **(A, C, E, G)** distributions of dendritic surface area - used as an approximation of excitatory synapse distributions - as a function of current transfer, and **(B, D, F, H)** current transfer as a function of path distance from the soma. Analyses were performed on normalized scales (left panels), where current transfer and path distance are expressed as percentages of their respective maxima, and on absolute scales (right panels). Apical (upper panels) and basal (lower panels) dendrites were analysed separately. Overall differences between UT and NT neurons were assessed using two-way ANOVA; significant differences are indicated by *p* values, non-significant differences correspond to *p* > 0.05, are not shown. Asterisks mark distance ranges or transfer intervals with significant differences between UT and NT neurons (Bonferroni *post hoc* test, *p* < 0.05). Distance regions and transfer intervals with fewer than three data points in either group were excluded from statistical analyses.

In the case of current transfer (Figure 4) three of eight graphs showed statistically significant overall US-induced alteration (two-way ANOVA, *p* < 0.001, location dependence over absolute and normalized scale in the basal dendrites and location dependence over absolute scale in the apical dendrites).

**Figure 5:**
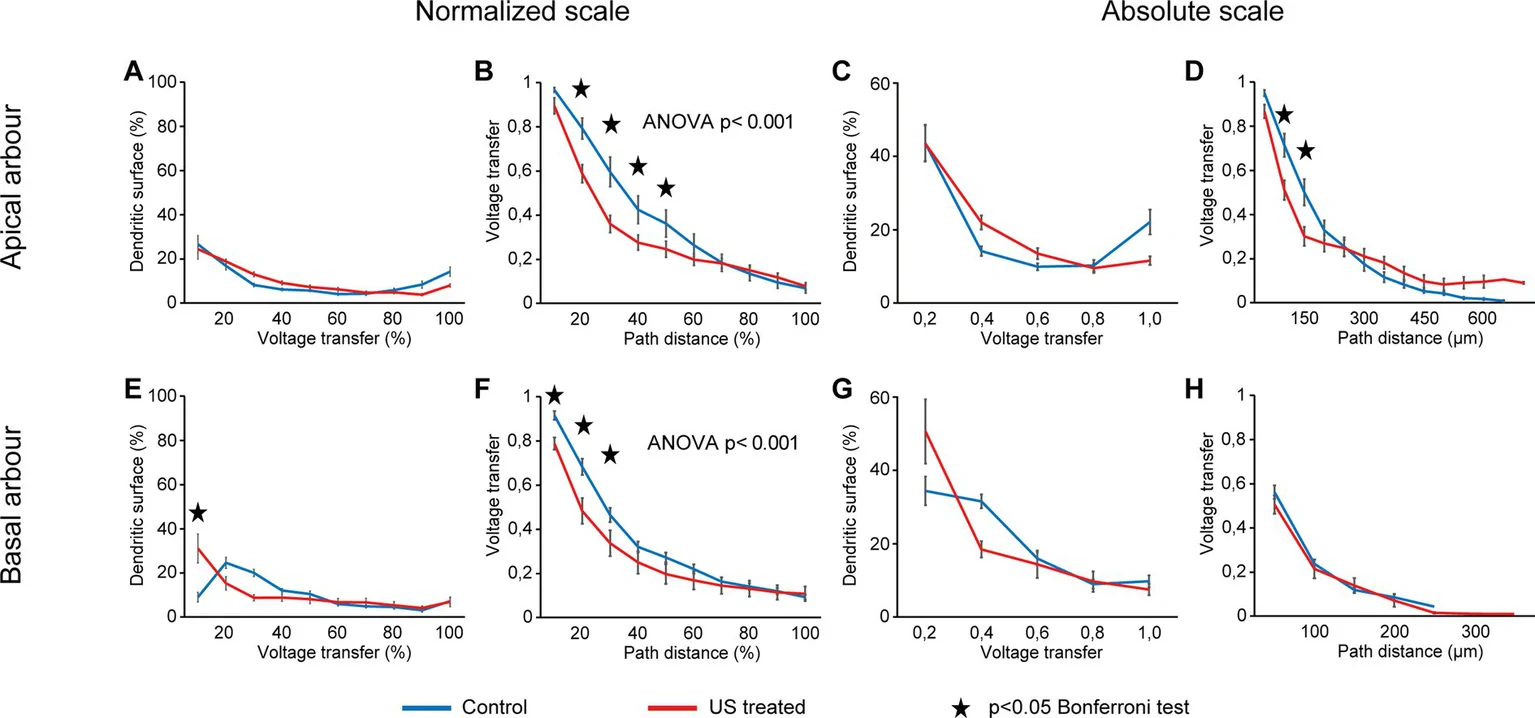
Altered steady-state voltage transfer of locally generated PSPs in passive models of UT CA1 pyramidal neurons. Comparison graphs illustrate alterations in steady-state voltage transfers of locally generated PSPs in UT (red) relative to control NT CA1 pyramidal neurons (blue). Two types of plots are shown: **(A, C, E, G)** distributions of dendritic surface area - used as an approximation of excitatory synapse distributions - as a function of steady-state voltage transfer, and **(B, D, F, H)** steady-state voltage transfer as a function of path distance from the soma. Analyses were performed on normalized scales (left panels), where steady-state voltage transfer and path distance are expressed as percentages of their respective maxima, and on absolute scales (right panels). Apical (upper panels) and basal (lower panels) dendrites were analysed separately. Overall differences between UT and NT neurons were assessed using two-way ANOVA, significant effects are indicated by *p* values, whereas non-significant differences correspond to *p* > 0.05, are not shown. Asterisks denote distance ranges or transfer intervals with significant differences between UT and NT neurons (Bonferroni *post hoc* test, *p* < 0.05). Distance regions and transfer intervals containing fewer than three data points in either group were excluded from statistical analyses.

**Figure 6:**
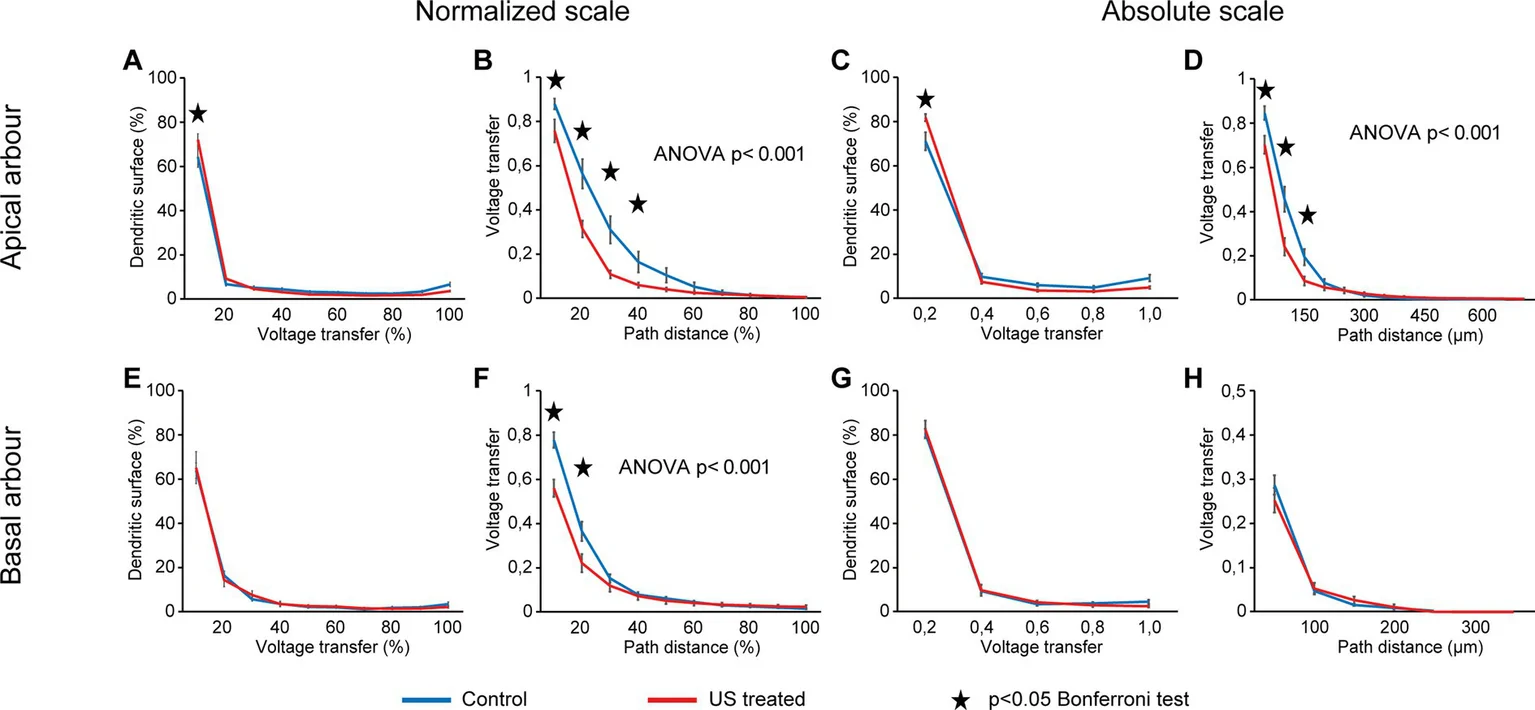
Alterations in sinusoidal voltage transfers of locally generated PSPs in passive models of UT CA1 pyramidal neurons. Comparison graphs illustrating changes in sinusoid voltage transfers in UT (red lines) neurons relative to control, NT neurons (blue lines). Two types of plots are shown: **(A, C, E, G)** distributions of dendritic surface area - used as an approximation of excitatory synapse distributions - as a function of sinusoidal voltage transfer, and **(B, D, F, H)** sinusoidal voltage transfer as a function of path distance from the soma. Analyses were performed on normalized scales (left panels), where sinusoidal voltage transfer and path distance are expressed as percentages of their respective maxima, and on absolute scales (right panels). Apical (upper panels) and basal (lower panels) dendrites were analysed separately. Overall effects were assessed using two-way ANOVA, statistically significant effects are indicated by *p* values, and whereas non-significant differences correspond to *p* > 0.05 are not shown. Asterisks denote path-distance ranges or transfer intervals in which UT and NT neurons significantly differed (Bonferroni *post hoc* test, *p* < 0.05). Distance regions and transfer intervals containing fewer than three data points in either group were excluded from statistical analysis.

As for the voltage transfers, two comparison graphs showed statistically significant overall alterations (two-way ANOVA *p* < 0.001) in steady-state voltage transfers (Figure 5) and three graphs in sinusoidal voltage transfers (Figure 6**).**

Statistically significant differences between UT and NT neurons were observed exclusively in the location dependence of current and voltage transfers; by contrast, the distribution of dendritic surface area as a function of transfers showed no such alterations (two-way ANOVA, *p* > 0.05).

In comparison graphs with distance dependence of current and voltage transfers, Bonferroni *post hoc* tests detected a few distance regions with significantly altered transfers in UT neurons. Distance regions, where generated PSPs had statistically different voltage transfers were always relatively close to the soma with distances not bigger than 50% of the maximum path distance. Further, current- and voltage transfers were always smaller in UT neurons relative to control in distance regions, where Bonferroni test detected significant alteration. Such tendency of decreased voltage and current transfers in UT neurons was present regardless of the type of scale used.

As we noted, the comparison graphs for the distributions of dendritic surface area (distribution of synapses) as a function of transfers did not show any statistically significant overall alteration in UT cells. However, Bonferroni *post hoc* tests showed a slight US-induced reorganisation of dendritic surface, a higher fraction of dendritic surface area (a higher fraction of synapses) is associated with lower transfers in UT neurons. Such structural reorganisation, coupled with the tendency of reduced transfers suggest that average transfer rates of PSPs elicited by a given pattern of simultaneous synaptic activities over the dendrites may be smaller in UT neurons. Therefore, these tendencies may lead to decreased general excitability of UT neurons.

### 4.5 Effects of US-induced morphological alterations on delays of PSPs in passive models

**Figure 7:**
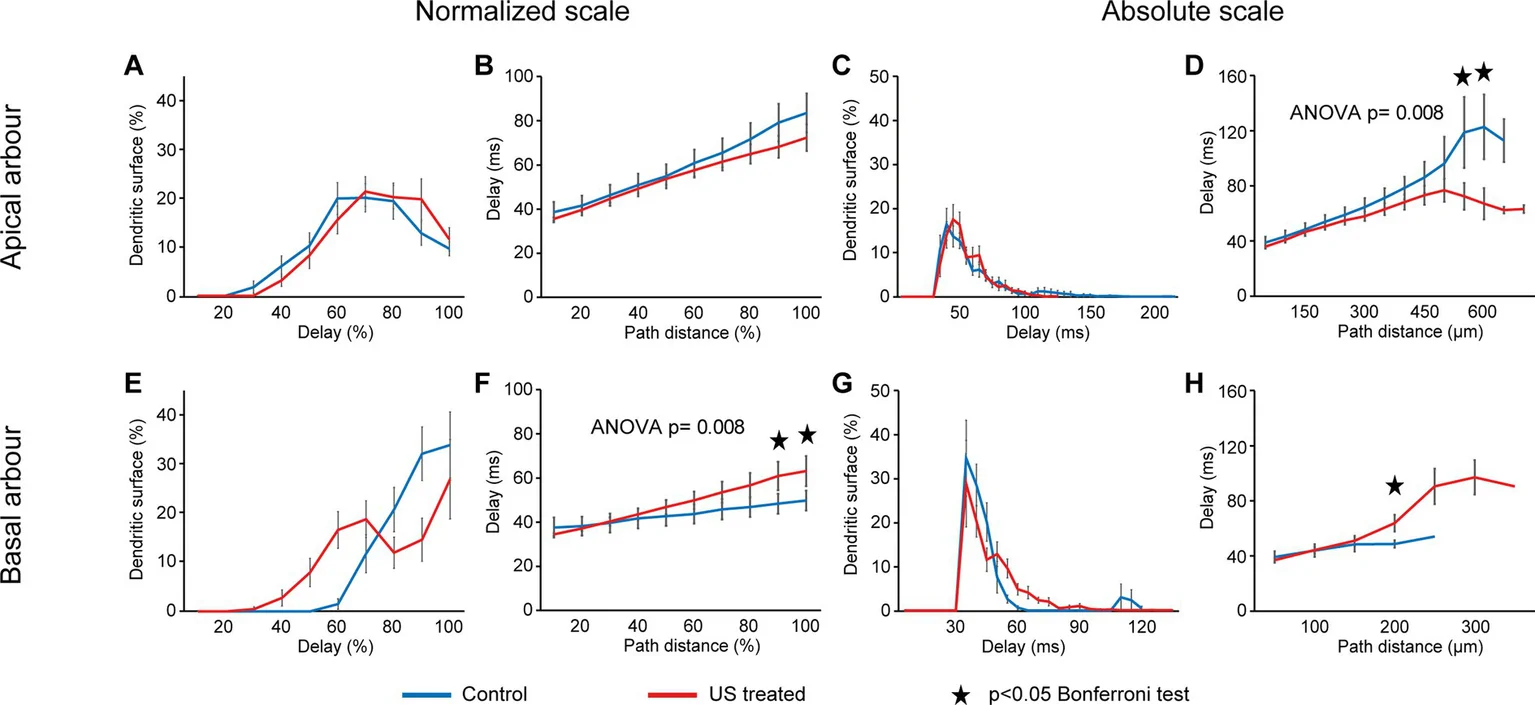
Alterations in delays of locally generated PSPs in passive models of UT CA1 pyramidal neurons. Comparison graphs illustrating changes in PSP delays in UT (red lines) neurons relative to control, NT neurons (blue lines). Two types of plots are shown: **(A, C, E, G)** distributions of dendritic surface area - used as an approximation of excitatory synapse distributions - as a function of delays, and **(B, D, F, H)** delays as a function of path distance from the soma. Analyses were performed on normalized scales (left panels), where delays and path distance are expressed as percentages of their respective maxima, and on absolute scales (right panels). Apical (upper panels) and basal (lower panels) dendrites were analysed separately. Overall effects were assessed using two-way ANOVA, statistically significant effects are indicated by *p* values, and whereas non-significant differences correspond to *p* > 0.05 are not shown. Asterisks denote path-distance ranges or transfer intervals in which UT and NT neurons significantly differed (Bonferroni *post hoc* test, *p* < 0.05). Distance regions and delay intervals containing fewer than three data points in either group were excluded from statistical analysis.

Distance dependence of delays showed statistically significant overall alterations on a normalized scale in the basal dendrites, whereas an absolute scale revealed changes in the apical dendritic arbours (Figure 7, two-way ANOVA, *p* = 0.008). The distributions of dendritic surface area as a function of delay showed no significant overall alteration. However, we observed a general, but, on an interval-wise basis, statistically rarely significant acceleration in dendritic signalling (decreased delays) within apical and a slowdown (increased delays) within basal dendrites (Figures 7C, D, G, H). These tendencies were consistently observed on absolute and normalized scales too. Such differential alterations in basal and apical dendritic delays suggest that speed of dendritic signal propagation may be differently affected by US treatment.

### 4.6 Effect of ultrasound on subthreshold signalling in active models

**Figure 8:**
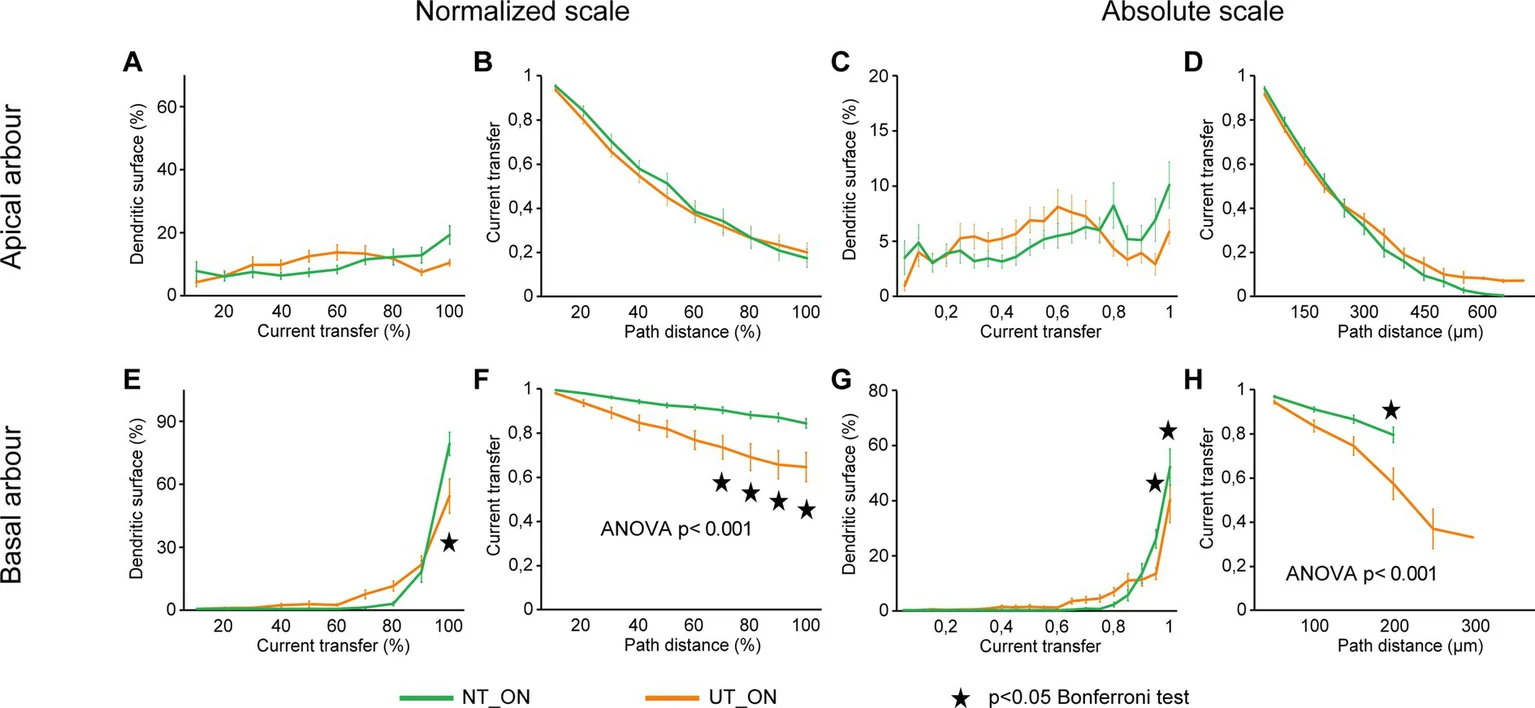
Altered current transfer of locally generated PSPs in active models of UT CA1 pyramidal neurons. Comparison graphs illustrate alterations in dendritic impulse propagation of UT (orange) relative to control NT (green) CA1 pyramidal neurons. Two types of plots are shown: **(A–G)** distributions of dendritic surface area - used as an approximation of excitatory synapse distributions - as a function of current transfer, and **(B–H)** current transfer as a function of path distance from the soma. Analyses were performed on normalized scales (left panels), where current transfer and path distance are expressed as percentages of their respective maxima, and on absolute scales (right panels). Apical (upper panels) and basal (lower panels) dendrites were analysed separately. Overall differences between UT and NT neurons were assessed using two-way ANOVA; significant differences are indicated by *p* values, non-significant differences correspond to *p* > 0.05, are not shown. Asterisks mark distance ranges or transfer intervals with significant differences between UT and NT neurons (Bonferroni *post hoc* test, *p* < 0.05). Distance regions and transfer intervals with fewer than three data points in either group were excluded from statistical analyses.

**Figure 9:**
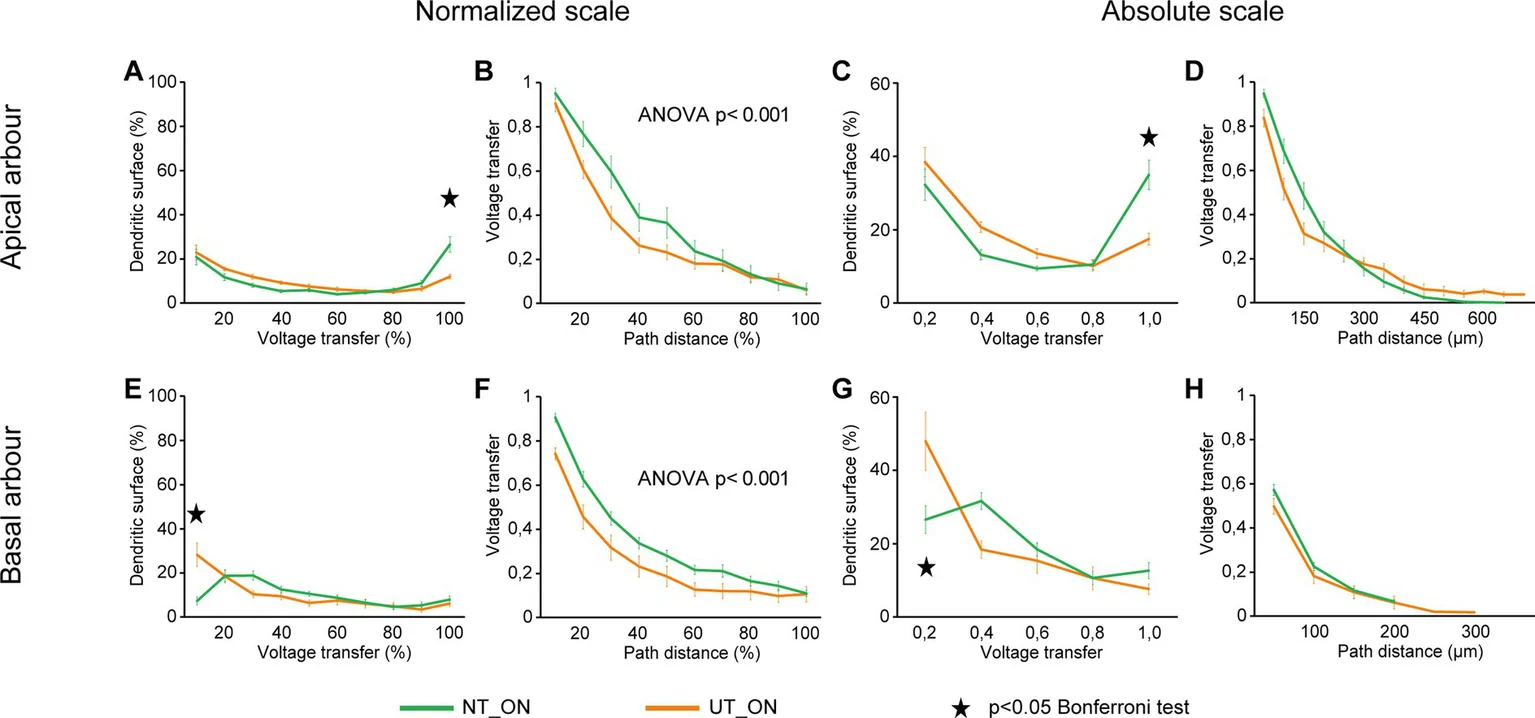
Altered steady-state voltage transfer of locally generated PSPs in active models of UT CA1 pyramidal neurons. Comparison graphs illustrate alterations in steady-state voltage transfers of locally generated PSPs in UT (orange) relative to control NT CA1 pyramidal neurons (green). Two types of plots are shown: **(A, C, E, G)** distributions of dendritic surface area - used as an approximation of excitatory synapse distributions - as a function of steady-state voltage transfer, and **(B, D, F, H)** steady-state voltage transfer as a function of path distance from the soma. Analyses were performed on normalized scales (left panels), where steady-state voltage transfer and path distance are expressed as percentages of their respective maxima, and on absolute scales (right panels). Apical (upper panels) and basal (lower panels) dendrites were analysed separately. Overall differences between UT and NT neurons were assessed using two-way ANOVA, significant differences are indicated by *p* values, whereas non-significant differences correspond to *p* > 0.05, are not shown. Asterisks denote distance ranges or transfer intervals with significant differences between UT and NT neurons (Bonferroni *post hoc* test, *p* < 0.05). Distance regions and transfer intervals containing fewer than three data points in either group were excluded from statistical analyses.

**Figure 10:**
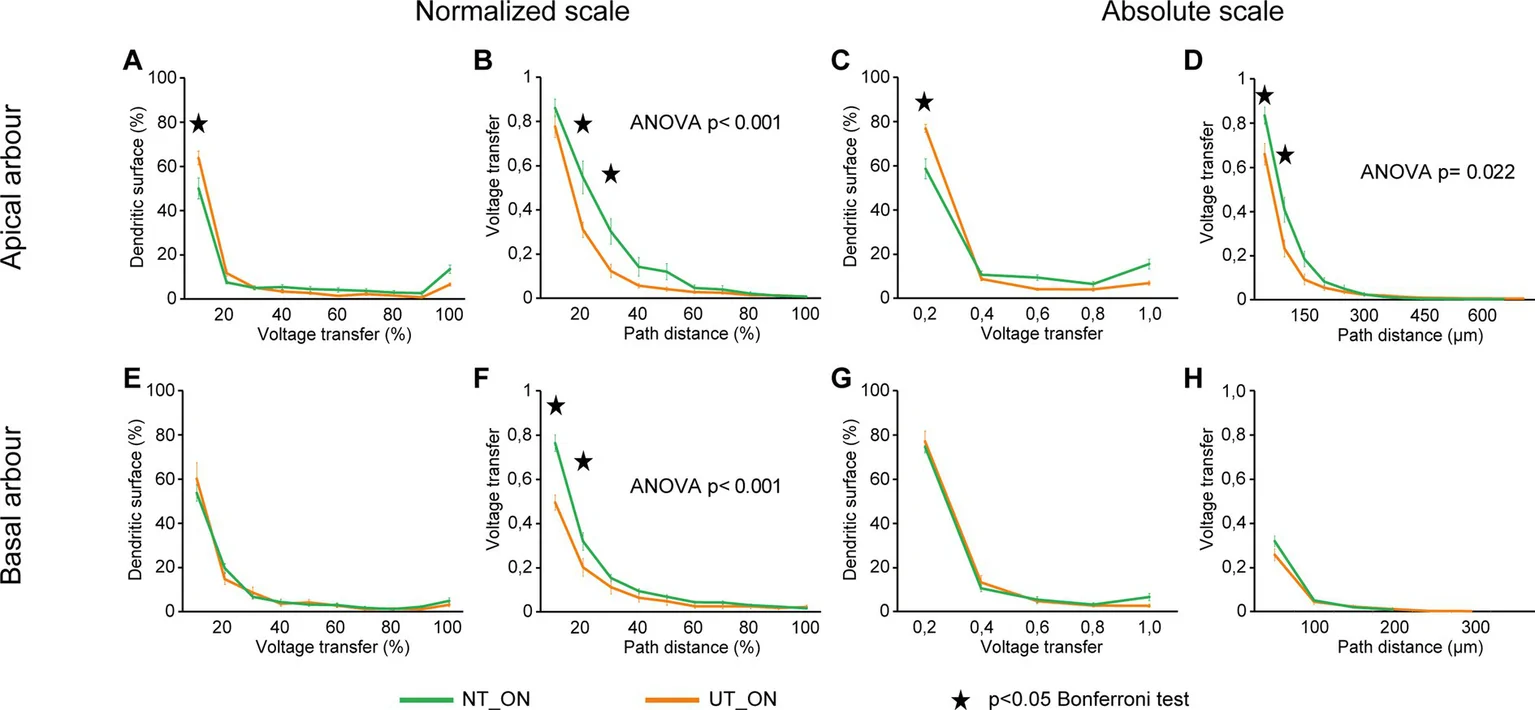
Alterations in sinusoidal voltage transfers of locally generated PSPs in active models of UT CA1 pyramidal neurons. Comparison graphs illustrate changes in sinusoid voltage transfers in UT (orange lines) neurons relative to control, NT neurons (green lines). Two types of plots are shown: **(A, C, E, G)** distributions of dendritic surface area - used as an approximation of excitatory synapse distributions - as a function of sinusoidal voltage transfer, and **(B, D, F, H)** sinusoidal voltage transfer as a function of path distance from the soma. Analyses were performed on normalized scales (left panels), where sinusoidal voltage transfer and path distance are expressed as percentages of their respective maxima, and on absolute scales (right panels). Apical (upper panels) and basal (lower panels) dendrites were analysed separately. Overall differences between UT and NT neurons were assessed using two-way ANOVA, significant differences are indicated by *p* values, whereas non-significant differences correspond to *p* > 0.05, are not shown. Asterisks denote distance ranges or transfer intervals with significant differences between UT and NT neurons (Bonferroni *post hoc* test, *p* < 0.05). Distance regions and transfer intervals containing fewer than three data points in either group were excluded from statistical analysis.

**Figure 11:**
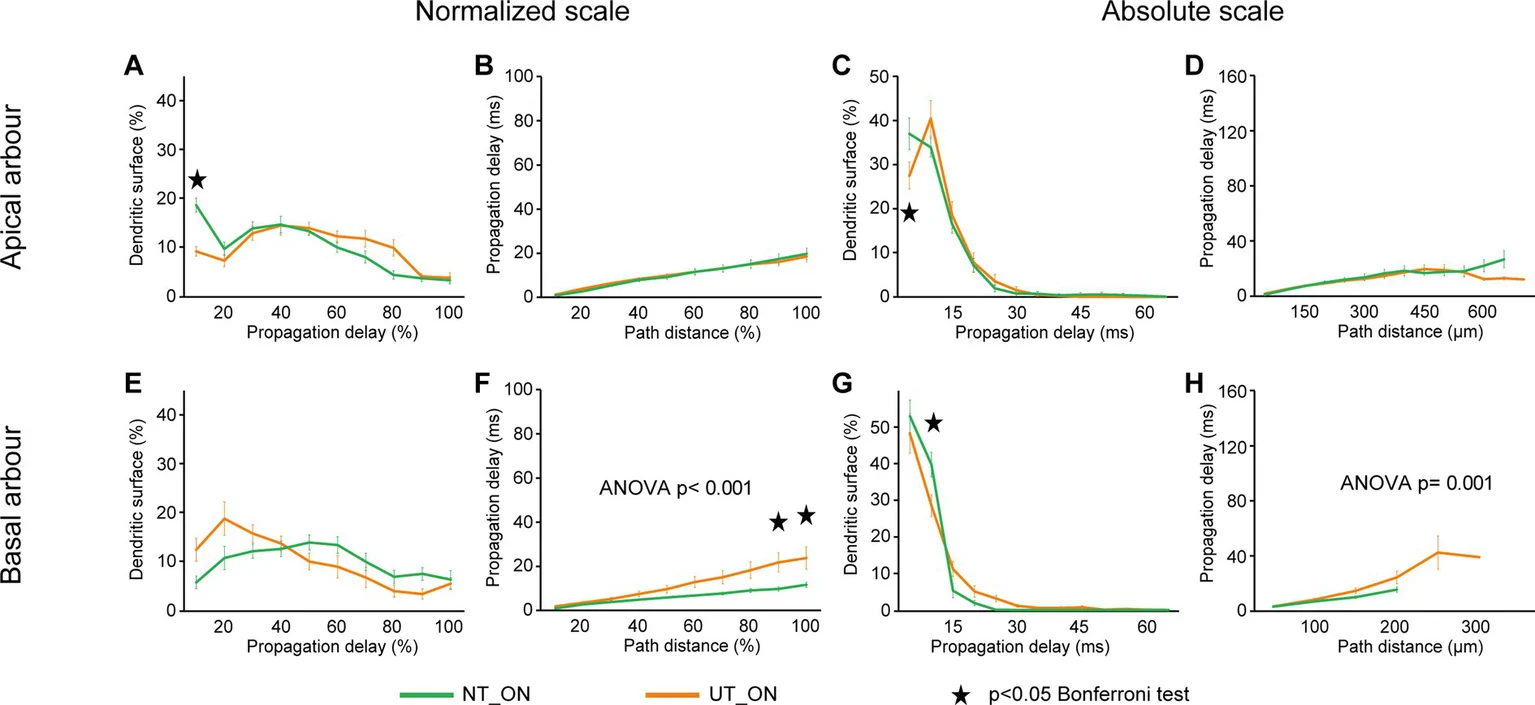
Alterations in propagation delays of locally generated PSPs in active models of UT CA1 pyramidal neurons. Comparison graphs illustrate changes in PSP propagation delays in UT (orange lines) neurons relative to control, NT neurons (green lines). Two types of plots are shown: (**A, C, E, G**) distributions of dendritic surface area - used as an approximation of excitatory synapse distributions - as a function of propagation delays, and (**B, D, F, H**) propagation delays as a function of path distance from the soma. Analyses were performed on normalized scales (left panels), where propagation delays and path distance are expressed as percentages of their respective maxima, and on absolute scales (right panels). Apical (upper panels) and basal (lower panels) dendrites were analysed separately. Overall differences between UT and NT neurons were assessed using two-way ANOVA, significant differences are indicated by *p* values, whereas non-significant differences correspond to *p* > 0.05, are not shown. Asterisks denote distance ranges or transfer intervals with significant differences between UT and NT neurons (Bonferroni *post hoc* test, *p* < 0.05). Distance regions and delay intervals containing fewer than three data points in either group were excluded from statistical analysis.

In this set of simulations, we analyzed the effects of ultrasound (US)-induced morphological alterations on subthreshold signalling in model neurons incorporating active HCN and K_A_ conductances (Figures 8–11).

#### 4.6.1 Dendritic receptive surface and synaptic efficiency

Analysis of dendritic surface area distributions, serving as a proxy for synaptic distribution, revealed that US treatment did not induce a significant overall functional rearrangement (Figures 8–11) in the apical or basal arbour (two-way ANOVA, *p* > 0.05).

#### 4.6.2 Current transfer

US treatment induced an overall decrease in steady-state current transfer within the basal arbour. This attenuation was highly significant across both absolute and normalized path distances (two-way ANOVA, *p* < 0.001 for both scales). Conversely, no such significant overall reduction in current transfer was detected in the apical arbour (Figure 8).

#### 4.6.3 Voltage transfer dynamics

Both steady-state (Figure 9) and sinusoid voltage (Figure 10) transfer efficacies were significantly modulated by US treatment, with effects localizing predominantly to the proximal regions of the apical arbour and throughout the basal arbour. Where point-by-point alterations reached statistical significance (Bonferroni *post hoc* test, *p* < 0.05), they consistently manifested as functional attenuations (decreases). Apical sinusoid voltage transfers underwent significant systemic alterations across both absolute (two-way ANOVA, *p* = 0.022) and normalized (two-way ANOVA, *p* < 0.001) path distances. In the basal arbour, US-induced alterations in sinusoid voltage transfer manifested significantly only across the normalized distance scale (two-way ANOVA, *p* < 0.001). The steady-state voltage transfers in both the apical and basal arbours exhibited significant overall alterations exclusively over normalized distance scales (two-way ANOVA, *p* < 0.001 for both arbours), while absolute scale distributions remained statistically unaffected.

#### 4.6.4 Propagation delays

Subthreshold signal propagation delays (Figure 11) were substantially prolonged by US exposure across the basal dendritic tree, with alterations detectable on both absolute and relative distance scales (two-way ANOVA, *p* < 0.001 for both scales). While this overall increase in latency was observable across all distance regions, it reached point-by-point significance (Bonferroni post hoc test, *p* < 0.05) only in the distal segments over the normalized scale. In contrast, within the apical arbour, no statistically significant overall or point-by-point alteration was detected.

### 4.7 Differential US-induced alterations in dendritic signalling of neurons

**Figure 12:**
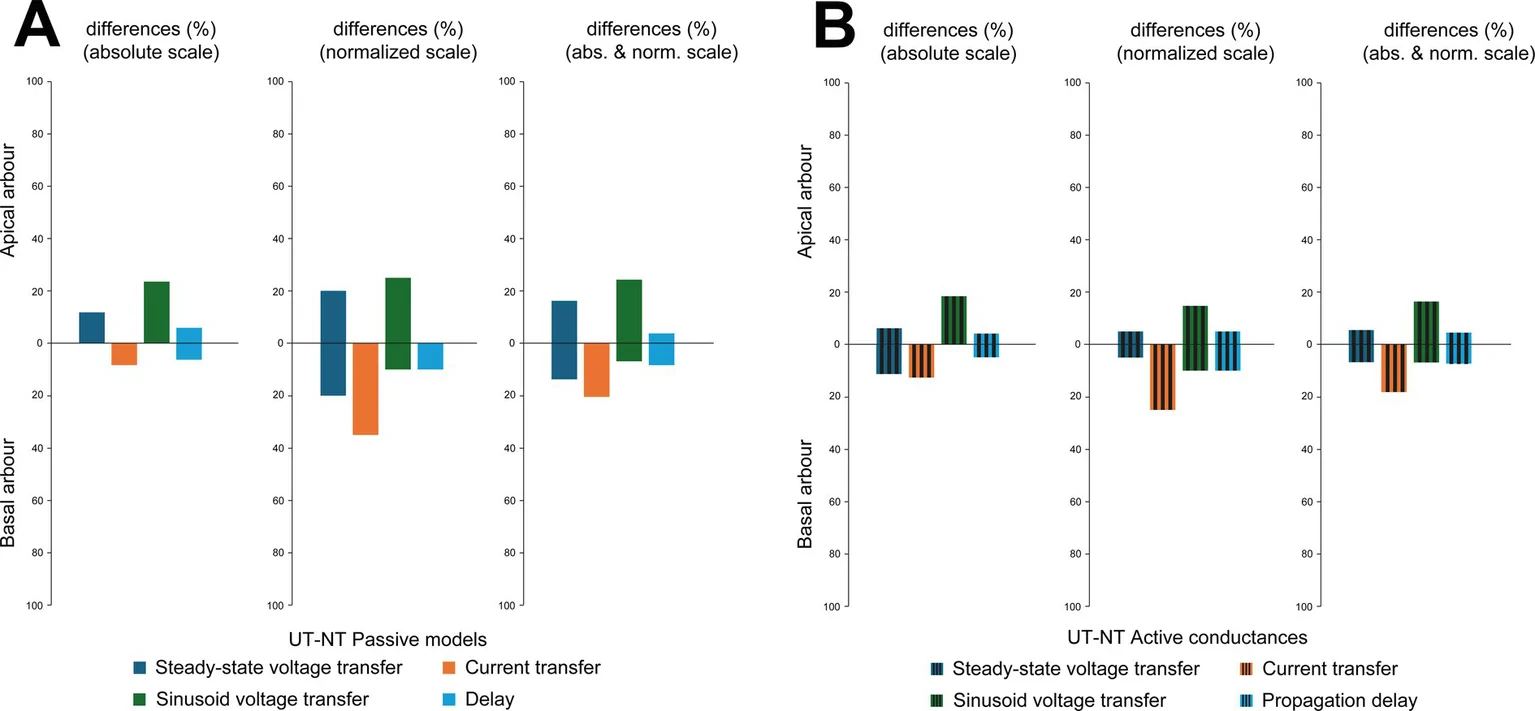
Summary of US-induced alterations in dendritic signalling of CA1 pyramidal neurons with passive and active membranes. Percentage of intervals showing significant alterations in dendritic signalling in UT neurons across absolute and normalized comparison scales, as well as a combined summary of alterations detected on both scales. Upper and lower bar extensions represent apical and basal dendritic arbours, respectively. The data presented here are derived from the comparison graphs shown in Figures 4–11. **(A)** Shows the data when passive membrane was used, while **(B)** shows the findings between UT and NT neurons with active conductances. The total number of intervals with at least three data points for both NT and UT neurons was defined as 100%.

We quantified the extent of US-induced alterations in dendritic signalling of scanning US treated CA1 pyramidal neurons. Alterations were explored in two different membrane models of these neurons; in purely passive membrane models and in models where HCN and K_A_ channels were inserted into the membrane alongside the leakage channels. This analysis was based on comparison graphs of descriptors of passive dendritic signalling (Figures 4–7), and on comparison graphs of active subthreshold signalling (Figures 8–11) where intervals with statistically significant differences (Bonferroni test, *p* < 0.05) between UT and NT neurons were marked by asterisks. We counted the number of intervals showing significant difference between the UT and NT cells, then converted these counts to percentages, taking the total number of intervals where statistical analysis was performed (distance regions and transfer and delay intervals with fewer than three data points in either group of cells were excluded) as 100%. This percentage of intervals, where statistically significant difference was detected between UT and NT neurons was employed to quantify the degree of US-induced alteration. We performed these calculations for comparison graphs with absolute and normalized scales, and by combining the results along both scales (Figure 12). Degrees of US-induced alterations in CA1 pyramidal neurons were dependent on the descriptor considered and for the same descriptor, alterations tended to be different in the apical and basal dendritic arbours.

#### 4.7.1 Passive models reveal limited spatial disruption

In purely passive models, fitted to electrophysiological population mean somatic DC impedance and time constants using only uniformly distributed leakage channels, the spatial extent of significant functional alterations was restricted (Figure 12A). Within the apical arbour, significant differences were confined almost entirely to steady-state and sinusoid voltage transfers, which exhibited deviations in only ~15–25% of the analyzed intervals across combined scales. Notably, passive apical current transfer demonstrated zero significant deviation across all intervals. In the basal arbour, the most pronounced deviation occurred in current transfer when evaluated on the normalized scale, affecting roughly 35% of the spatial intervals. However, other basal signalling descriptors, such as delay and sinusoid voltage transfer, remained highly stable, showing significant localized alterations in only ~10% or less of their spatial reach.

#### 4.7.2 Active conductances further restrict functional deviations

When physiological active conductances (HCN and K_A_ channels) were included and fitted to the same somatic population means, the extent of US-induced alterations became even more profoundly restricted (Figure 12B). In the apical arbour of the active (ON) models, the proportion of significantly altered intervals for sinusoid voltage transfer dropped below 20% on the combined scale. Steady-state voltage transfer and propagation delays exhibited negligible spatial disruption (~5%), while current transfer remained entirely unperturbed (0%) across all scales. A similar buffering effect was observed in the active basal arbour: the extent of altered current transfer (on the normalized scale) narrowed to approximately 25%, with steady-state voltage transfer, sinusoid voltage transfer, and propagation delays showing significant deviations in only 5–11% of the evaluated intervals.

#### 4.7.3 Conclusion of spatial extent

In summary, these quantifications reveal that across both the apical and basal arbours, and regardless of the presence or absence of active conductances, the vast majority of the dendritic tree’s functional territory remains statistically identical to control (NT) cells. Typically, 75–100% of the analyzed intervals for any given signalling descriptor showed no significant deviation. Therefore, we conclude that diagnostic ultrasound exposure does not affect intradendritic subthreshold signalling in a spatially widespread or systemic manner. Instead, functional alterations are highly localized and spatially restricted, allowing the neuron to fundamentally preserve its overall computational architecture and signalling integrity.

### 4.8 Regulatory role of active conductances in UT models

#### 4.8.1 Steady-state spatial transfer enhancement in the passive state

To isolate the functional contribution of subthreshold active conductances in ultrasound-treated neurons, we compared steady-state and dynamic signal transmission in UT cell models with HCN and K_A_ channels active (UT_ON) versus deactivated (UT_OFF, Supplementary Figures 1–4). Deactivation of these active conductances significantly enhanced both steady-state current and voltage transfers across the dendritic arbours. In the apical arbour, the transition to the passive UT_OFF state resulted in highly significant overall increases in current transfer and steady-state voltage transfer (two-way ANOVA, *p* < 0.001 for both). A similar significant enhancement was observed in the basal arbour for both current (two-way ANOVA, *p* = 0.003) and steady-state voltage transfers (two-way ANOVA, *p* = 0.014). This indicates that the resting parallel leak introduced by HCN and K_A_ channels heavily attenuates spatial signal integration, and their removal substantially extends the effective length constant of the UT dendrites.

#### 4.8.2 Propagation delays and temporal synchronization

While steady-state spatial transfer improved in the passive model (Supplementary Figures 1, 2), this came at a severe temporal cost. Propagation delays were drastically prolonged in the UT_OFF models (Supplementary Figure 4) across both arbours (two-way ANOVA, *p* < 0.001 for both apical and basal regions). In the apical arbour, this prolongation was highly distance-dependent, driven by the distal accumulation of *I_h_* channels. Upon deactivation, distal apical delays increased nearly four-fold, jumping from roughly 15–20 ms in the UT_ON state to 60–80 ms in the UT_OFF state. This profound temporal shift highlights the critical homeostatic role of active conductances; they act as an indispensable temporal governor in UT cells, aggressively accelerating subthreshold signal propagation to maintain input–output synchronization despite the cell’s underlying morphological alterations.

#### 4.8.3 Stability of high-frequency transfer

In stark contrast to steady-state transfers and propagation delays, 50 Hz sinusoid (dynamic) voltage transfer remained universally unaffected by the deactivation of HCN and K_A_ channels (Figure 3). The dynamic transfer attenuation curves for the UT_ON and UT_OFF states were virtually superimposable across both absolute and normalized scales in the apical and basal arbours. This structural stability demonstrates that the severe attenuation of high-frequency inputs in UT cells is driven strictly by passive capacitive shunting. The high-frequency gamma-band signals are heavily filtered by the global membrane capacitance, a passive mechanism that is completely insensitive to the modulation of relatively slow-gating subthreshold active conductances.

### 4.9 Synaptic input pattern recognition remains unaltered in US treated CA1 pyramidal neurons

**Figure 13:**
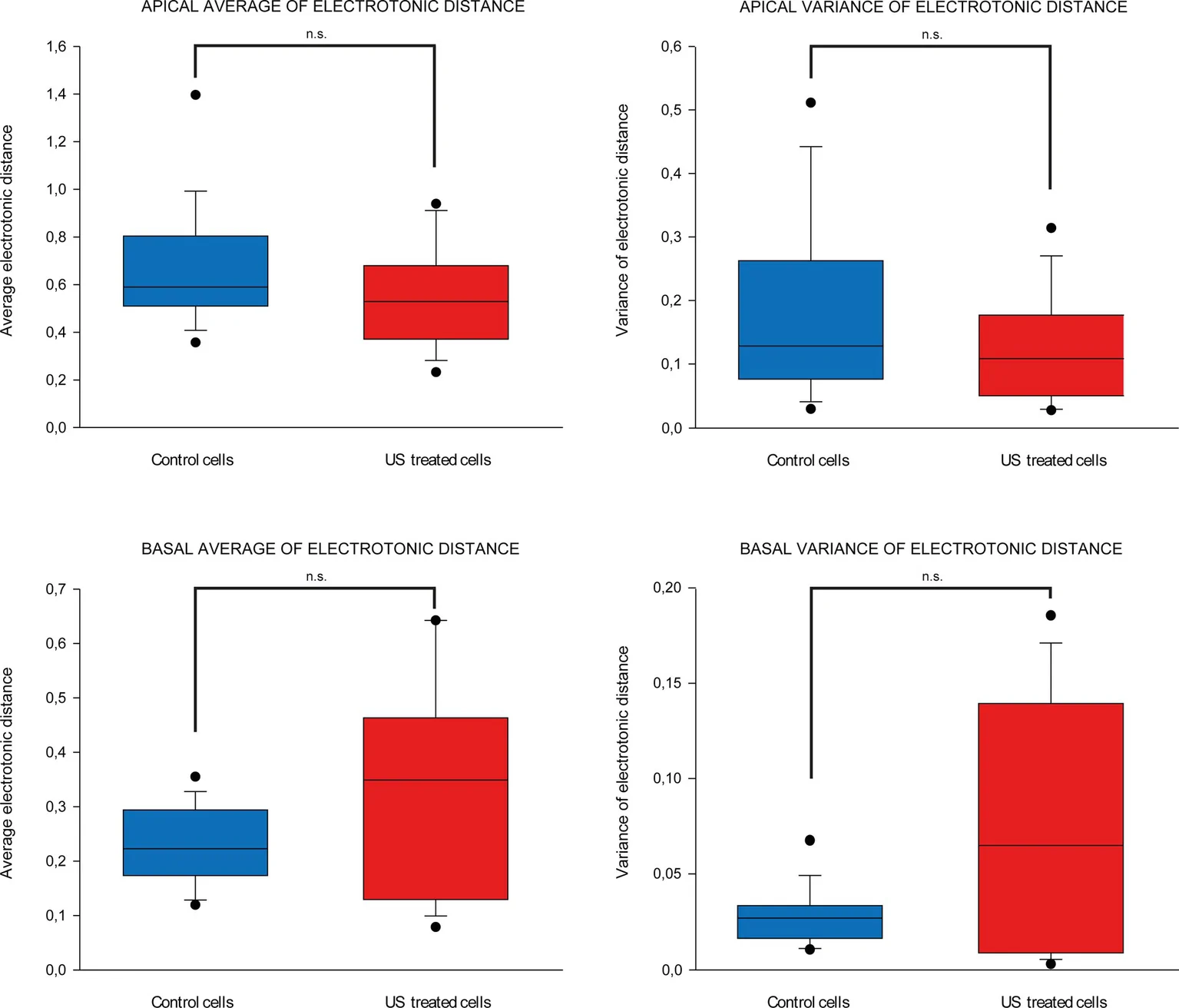
Synaptic input pattern recognition in apical and basal dendrites of NT and UT hippocampal pyramidal neurons. Predictors of synaptic input pattern recognition and discrimination remained unchanged in UT CA1 hippocampal neurons (Mann–Whitney test: *p* > 0.22, *p* > 0.21, *p* > 0.09, *p* > 0.08 for apical mean and variance and basal mean and variance of electrotonic distances, respectively). Means and variances of electrotonic distances of simulated synaptic inputs (injection sites) from the soma were used to quantify and compare pattern recognition/discrimination capacity in NT (blue) and UT (red) neurons. Apical (upper panels) and basal (lower panels) dendritic arbours were analyzed separately. Boxes indicate the 25th and 75th percentiles; the central line denotes the median and whiskers represent the minimum and maximum values.

Previously, we examined the effects of US treatment on dendritic propagation of a single, locally generated PSP. *In vivo*, however, neurons are exposed to multiple synaptic inputs that overlap in time and summate at the axon initial segment, thereby shaping neuronal firing. The ability of neurons to distinguish between distinct spatial patterns of synaptic activation is a key feature of information processing and underlies cognitive functions such as learning and memory. Accordingly, we investigated whether repeated diagnostic-level US exposure alters synaptic input pattern recognition in CA1 pyramidal neurons. Our analysis was based on the framework established by de Sousa et al. (2015), who found that two predictors; the mean and variance of electrotonic distances between synapses and the soma correlate with input pattern recognition/discrimination in neurons with both active and passive membranes. We observed no significant differences in these predictors of synaptic input pattern recognition of UT and control neurons (Figure 13). Specifically, neither the mean nor the variance of electrotonic distances differed significantly in apical or basal dendrites (Mann–Whitney test: *p* > 0.22, *p* > 0.21, *p* > 0.09, *p* > 0.08 for apical mean and variance and basal mean and variance of electrotonic distances, respectively).

## 5 Discussion

Prior studies have shown that US increases the expression of neuronal activity-associated markers such as BDNF and c-Fos, both of which are known to regulate dendritic spine density and morphology (Duque et al., 2022; Papp et al., 2022). Structural modifications of the dendritic arbour have been demonstrated to substantially influence burst firing in pyramidal neurons and, in some cases, exert a stronger effect than changes in membrane conductance alone (van Elburg and van Ooyen, 2010; Poirazi and Papoutsi, 2020). Moreover, multiple studies indicate that US can either potentiate or suppress neuronal firing depending on the applied stimulation parameters (Blackmore et al., 2019). Consistent with this, Clennell et al. (2021) reported that US can induce reversible, plastic-like modifications in neuronal circuits. Taken together, these findings support the hypothesis that morphological and electrophysiological alterations observed in UT CA1 pyramidal neurons may arise from the cumulative effects of repeated, transient plasticity-like changes triggered by US exposure. At the same time such alterations are likely to be partially offset by compensatory mechanisms, potentially limiting their net biological effect at the cellular or system level. In this context, we aimed to determine whether the previously identified morphological changes in UT neurons (Winkler-Ferenczi et al., 2024) translate into significant alterations in their synaptic input pattern recognition capacity.

de Sousa et al. (2015) have shown that both the mean and the variance of synaptic electrotonic distances are valid predictors of synaptic input pattern recognition and discrimination in neurons with either active or passive membranes. Our analysis revealed no statistically significant differences in these predictors between US-treated and control CA1 pyramidal neurons. While direct experimental data on the possible effects of scanning US on active membrane conductances are currently unavailable, conservation of integrative properties suggest that such conductances, if altered at all, do not deviate significantly from the physiological range of control neurons.

### 5.1 Effects of ultrasound-induced morphological alterations on dendritic signalling

Our simulations predicted no significant alteration in specific membrane resistance and capacitance within UT hippocampal CA1 pyramidal neurons. On the other hand, (Winkler-Ferenczi et al., 2024) described that diagnostic ultrasound exposure induces localized shifts in dendritic morphology of these neurons. These structural modifications included significant segment elongation and elevated spine density in the basal arbour. In the apical arbour of UT neurons, the combined effect of decreased spine density and reduced average spine surface area leads to a reduction in the spine surface area contribution per micrometer of dendrite length to one-third of the control. In the basal arbour the spine density increase overweighted the small decrease in spine surface area and eventually led to an increase in spine surface area contribution in this part of the neuron. Furthermore, morphometric analysis indicated that UT neurons exhibit a significantly decreased soma surface area relative to their NT counterparts.

Because our computer models faithfully captured all these morphological changes and predicted passive membrane properties (*R_m_* and *C_m_*) to remain conserved in UT and NT cells, any observed alterations in dendritic signalling within passive cable models of UT neurons can be entirely attributed to the complex interplay of the morphological alterations. Given the multi-parametric nature of these morphological variations, it is crucial to isolate how each individual morphological feature shapes signal propagation independently of concurrent structural effects.

*Somatic surface area* is inversely proportional to the somatic input resistance. Consequently, the reduced soma surface area in UT neurons inherently increases the input resistance of the somatic compartment by decreasing its total leak conductance. This will reduce the “sink” effect characteristic of larger somata. As a result, a smaller fraction of the current injected to the dendrite will flow towards the soma and a larger portion will leak through the dendritic membrane leading to a reduction of electrical charge that eventually reaches the cell body. Thus, a reduction in the soma size inherently decreases current transfer to the cell body when all other geometric parameters remain the same. Ultimately, higher somatic input resistance allows that even tiny synaptic currents can generate more robust somatic voltage changes, whereas a massive somatic volume and surface area act as an electrical “sink,” requiring massive synaptic current to achieve the same shift in membrane potential.

The severe *pruning of apical spines*, which reduced the spine surface area contribution per micrometer to one-third of control values, exerts a profound influence on signal propagation. Because spines significantly contribute to the total dendritic surface area, this physical loss of membrane effectively removes parallel leak channels and capacitive loads from the apical dendritic compartments. Consequently, this pruning increases the local transverse membrane resistance and lengthens the electrotonic length constant. By limiting transmembrane current leak as signals travel toward the soma, this structural modification, assuming everything else remains unchanged, theoretically enhances both voltage and current transfers. In contrast, US-induced *spinogenesis in the basal arbour* exerts the opposite effect, increasing local membrane surface area, which decreases the transverse membrane resistance and shortens the length constant.

*US-induced current and voltage transfer alterations* in the passive models can be entirely understood through this interplay of somatic and dendritic modulatory effects.

In the basal arbour, current transfer significantly decreased because the diminished “pulling” (sink) effect of the shrunken soma compounded with the increased dendritic leak introduced by spinogenesis. Apically, the reduced somatic sink effect was counteracted by the massive spine pruning, which reduced dendritic current leak; these opposing forces effectively canceled each other out, resulting in unchanged current transfer in the proximal apical dendrites.

Steady-state voltage transfer remained unaffected in the basal arbour because the reduction in current reaching the soma was perfectly offset by the higher somatic input resistance, which preserved the somatic voltage response. However, the dynamics in the apical arbour of UT cells highlight the dominance of the dendritic alterations. While the equivalent current reaching the smaller soma generates a larger somatic voltage shift (V_soma_), this effect is vastly overcompensated by the massive US-induced pruning of apical dendrites. This pruning drastically increases the local transverse membrane resistance, causing the local dendritic voltage response (V_dend_) to elevate. Because voltage transfer is calculated as the ratio of somatic to local dendritic voltage (V_soma_/V_dend_), the disproportionate rise in local dendritic voltage drives the proximal voltage transfer down in UT neurons.

Finally, increased *elongation of dendritic segments combined with an increased branching tendency* within the basal arbour of UT neurons compromise somatopetal signal transfer while increasing propagation delays, assuming no other change in morphology. This is because segment elongation leads to increased delays over larger geometrical distances and, due to the increased membrane surface along the longer path, increasing leaking currents reducing transfers. Each bifurcation introduces a potential impedance mismatch. When a voltage signal travels from a thin distal dendritic tip toward the soma (proximal direction), it hits a node where it encounters the sister branch and the parent segment. Every additional branch added to the dendritic tree introduces more membrane surface area, which scales up the total dendritic capacitance. When a transient electrical wavefront reaches a bifurcation, it must charge not only the forward path but also the capacitance of all attached sister structures. This collective capacitive loading slows down the charging rate (increases the rise time) of the propagating wave, generating longer propagation delays.

By combining classical cable theory equations (Rall, 1959; Jack et al., 1975; Tuckwell, 1988; Major et al., 1993) with a simple ball-and-stick approach and expanding it to two idealized dendrites (apical and basal) attached to a soma (Coombes et al., 2007; Timofeeva et al., 2008), one can derive analytical expressions that capture the principal trends in signal alterations observed in our simulations.

Among the principal expressions, the steady-state voltage transfer ratio ( $X_{V}$ ) from the apical dendrite (Dendrite 1) to an effective somatic conductance ( $G_{S,\mathrm{eff}}$ ) is expressed as:


$$A_{V,1}(X_{1})=\frac{1}{\cosh(X_{1})+(\frac{G_{S,\mathrm{eff}}}{G_{\infty ,1}})\sinh(X_{1})}$$

Similarly, the frequency-dependent AC length constant (λ_AC_) and the somatopetal centroid propagation delay (τ_cent_) over a physical distance x can be analytically approximated as:


$$\begin{array}{l} \lambda_{\mathrm{AC}}(\omega )=\lambda \sqrt{\frac{2}{\sqrt{1+{\left(\omega \tau_{m}\right)}^{2}}+1}}\\ \tau_{\text{cent}}(x)\approx \frac{r_{a}c_{m}}{2}x^{2}+r_{a}c_{m}x(\frac{G_{\text{sink}}}{G_{\infty }}) \end{array}$$

where $X_{1}$ is the dimensionless electrotonic distance, $G_{\infty }$ is the characteristic conductance, ω is the angular frequency, $\tau_{m}$ is the membrane time constant, $c_{m}$ is the membrane capacitance per unit length, and $r_{a}$ is the axial resistance. These expressions provide an analytical framework for interpreting how isolated and combined US-induced morphological shifts, such as soma shrinkage $(G_{S,\mathrm{eff}}$ ↓), segment elongation ( x ↑) or spine pruning ( $c_{m}$ ↓, $G_{\infty }$ ↓), interact to produce the signalling alterations we observed. Full derivation and quantitative illustrations of these formulas are presented in the Supplementary note.

### 5.2 Isolation of the functional role of HCN and K_A_ channels in UT neurons

By utilizing models of UT neurons with active major voltage gated ion channels operating in the subthreshold state (HCN and K_A_ channels ON) fitted to experimental somatic parameters, we isolated the regulatory role of these conductances by subsequently deactivating them (OFF state). The removal of active conductances significantly improved steady-state voltage and current transfers across the entire dendritic arbour, reflecting the elimination of the resting conductance for *I_h_* current and a subsequent increase in the membrane length constant. However, this spatial enhancement came at a severe temporal cost.

In the passive (OFF) state, propagation delays were drastically prolonged, as removal of HCN and K_A_ channels increased membrane resistance, ultimately increasing the membrane time constant. Consequently, the activation of *I_h_* channels acts as a critical homeostatic equalizer: the massive resting leak drastically lowers the local membrane resistance, shrinking the time constant and forcing rapid signal propagation. Thus, our results demonstrate that active HCN and K_A_ conductances in UT cells actively override one consequence of US-induced spine pruning (the loss of leak), ensuring temporal synchronization of distal inputs at the expense of severe spatial attenuation.

In stark contrast to the steady-state transfers, the 50 Hz dynamic voltage transfer curves for the ON and OFF states are virtually identical because of the capacitive shunting. At high frequencies (like 50 Hz), the membrane capacitor provides a very low-impedance path for the signal to escape. This capacitive shunting is so powerful that it completely dominates the local impedance. Because the active conductances (*I_h_* and *I_A_*) primarily affect the resistive component of the membrane impedance and have relatively slow gating kinetics, their presence or absence makes almost no difference to a fast 50 Hz AC signal. The capacitance is the sole dictator of high-frequency attenuation, which is why turning the channels OFF does not alter the 50 Hz transfer.

In summary, HCN and K_A_ channels act as complex functional modulators in the subthreshold signalling; speed up propagation of dendritic signals, weaken steady-state transfers but do not alter high frequency (50 Hz) voltage transfer.

### 5.3 Rationale for selecting HCN and A-type potassium channels

While our computational framework successfully evaluates how ultrasound-induced morphological changes alter subthreshold dendritic integration, it is important to acknowledge the boundary conditions of our active models. Specifically, we restricted voltage-dependent conductances to hyperpolarization-activated cyclic nucleotide-gated (HCN) and A-type potassium (K_A_) channels. The primary rationale for selecting these channels is their dominant operational voltage range and precise anatomical distribution. HCN channels are partially open at rest, dictating baseline input resistance and membrane time constants, whereas K_A_ channels operate at subthreshold depolarizations to regulate the amplitude and duration of incoming excitatory postsynaptic potentials (EPSPs). Importantly, the expression densities of both channels scale significantly with distance from the soma in CA1 pyramidal cells (Noh et al., 2019; Masurkar et al., 2020). Because ultrasound exposure induces arbour-specific geometric variations, these morphological shifts alter the effective number of active channels in a location-dependent manner, thereby changing signal transfers and propagation delays in ways that a purely passive model cannot capture. Other voltage-gated channels generally lack these precise somato-dendritic gradients tuned specifically for subthreshold spatial filtering.

Furthermore, omitting channels primarily associated with suprathreshold events (such as voltage-gated sodium, high-voltage activated calcium, and delayed rectifier potassium channels), is a standard and necessary simplification in this context (Li et al., 2021). Because our study exclusively measures the transfers and delays of subthreshold postsynaptic potentials, these high-threshold channels would largely remain in their closed states during the simulated events. While certain secondary subthreshold currents (such as T-type calcium or persistent sodium channels) could theoretically modulate signal transfer, their inclusion would introduce a highly non-linear, multi-dimensional parameter space, adding computational overhead without meaningfully altering the fundamental subthreshold voltage trajectories (Migliore et al., 1999). By deliberately omitting these additional currents, we prioritized model tractability and minimized the risk of parameter unidentifiability, a well-documented limitation in multicompartmental modeling where an over-parameterized model can produce identical voltage trajectories through non-biological combinations of active conductances (Prinz et al., 2004; Keren et al., 2005). This constraint cleanly isolated how ultrasound-altered dendritic geometry interacts with the primary active filters (HCN and K_a_) to govern spatial attenuation.

## 6 Conclusion

**Figure 14:**
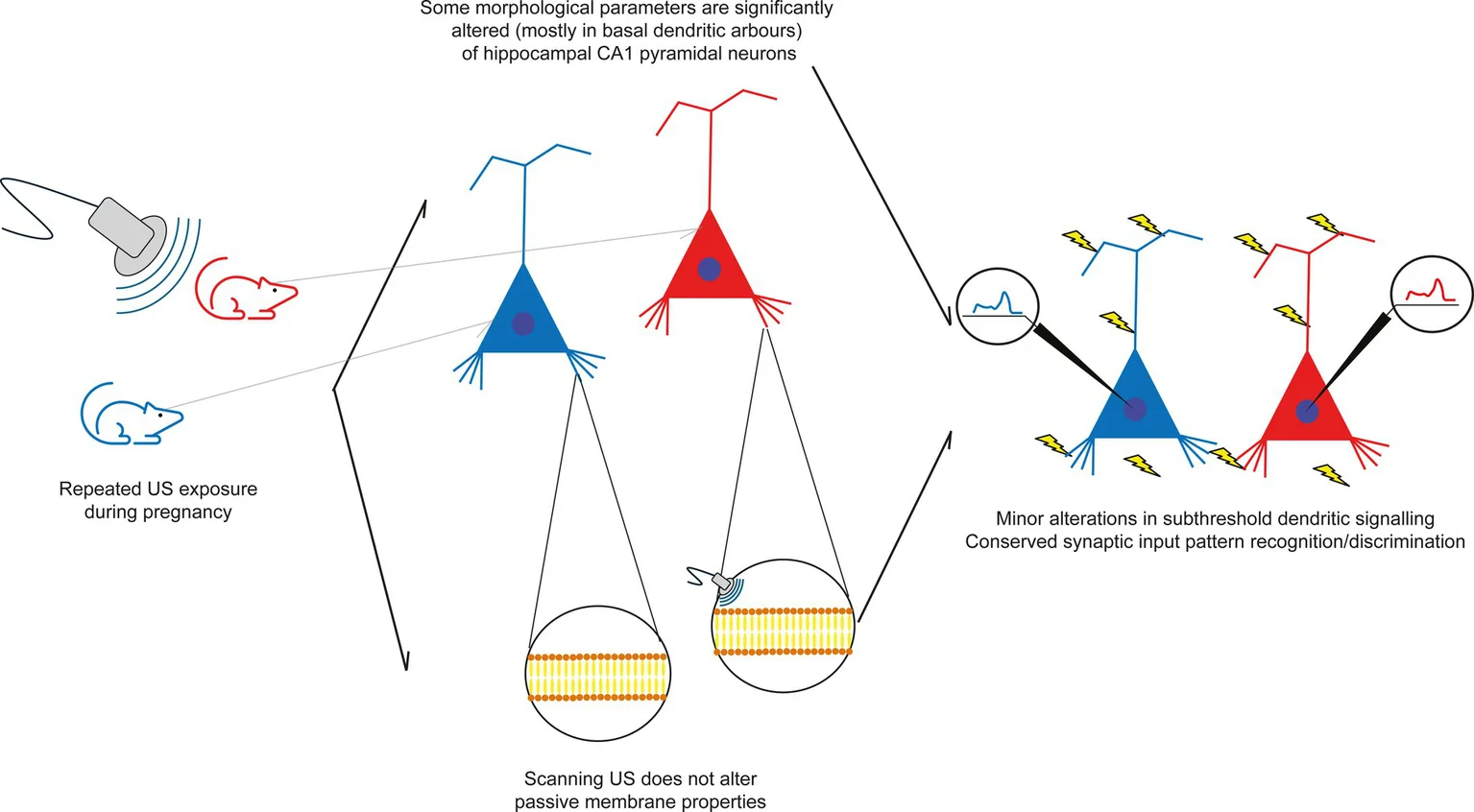
Summary: Morphofunctional effects of scanning US on CA1 hippocampal pyramidal neurons. US alters the morphology of dendrites, meanwhile passive membrane properties, such as specific membrane resistance and capacitance remain unchanged. Our computer simulations revealed that intradendritic signalling underwent minor alterations, as measured by steady-state and sinusoidal voltage and current transfers, as well as by delays. Notably, these alterations were observed in PSPs originating from only a fraction of synapses. Such limited alterations in transfers and delays of PSPs may leave synaptic integration unchanged, leading to unaltered input pattern recognition/discrimination in UT neurons. NT neurons in blue, UT neurons in red colours.

The present study investigated the effects of repeated diagnostic-level US on CA1 pyramidal neurons in 28-day-old mice. Using morphologically faithful subthreshold passive and active segmental cable models of these cells, we analysed somatopetal dendritic signal propagation, focusing on the attenuation and delay of postsynaptic potentials. Ultrasound treatment is not just causing morphological alterations; it is triggering a mild complex, asymmetric (apical vs. basal) active structural reorganization of the neuron. As a consequence of US-induced morphological modifications, minor alterations were found in PSP attenuation, and delays, which are modulated further when HCN and K_A_ channels were added to model neurons. However, quantitative predictors of synaptic input pattern recognition were not affected (Figure 14). Taken together, these findings indicate that repeated scanning US treatment does not substantially impair the intradendritic signalling or integrative properties of CA1 pyramidal neurons within our experimental framework. While extrapolation to humans must be made with caution, our results support the notion that diagnostic-level US is unlikely to exert substantial effects on intradendritic signal processing during prenatal exposure.

## 7 Limitations

Due to the lack of detailed information on US-induced alterations in distribution and density of active ion channels following repeated (scanning) US exposure, our simulations were restricted to subthreshold passive and active membrane models and we did not attempt to address suprathreshold events. Importantly, the predictors of synaptic input pattern recognition used here are applicable to neurons with both passive and active dendritic membranes.

## Data Availability

The datasets presented in this study can be found in online repositories. The names of the repository/repositories and accession number(s) can be found below: The [.hoc]hoc code for the simulation of dendritic signalling is shared on at: https://github.com/somogyia/Intraneuronal-dendritic-signalling. The underlying data sets of reconstructed neurons and the large amount of raw data of simulations are available from the corresponding author on request.
