# Supplementary material for: Effects of repetitive scanning ultrasound on the intraneuronal dendritic signalling of CA1 pyramidal neurons
Source: Front Behav Neurosci. 2026 Aug 7;20:1871826. doi: 10.3389/fnbeh.2026.1871826 (PMC13493503; doi:10.3389/fnbeh.2026.1871826)
Supplement: Supplementary file 1 [file Data_Sheet_1.DOCX]

**Supplementary Figure 1.** Contributions of subthreshold active conductances to current transfer in ultrasound treated CA1 pyramidal neurons with HCN and K_A_ channels active (UT_ON) versus deactivated (UT_OFF).
Comparison graphs illustrate alterations in dendritic impulse propagation in UT neurons with activated channels (orange) relative to UT neurons with deactivated channels (red). Two types of plots are shown: (A, C, E, G) distributions of dendritic surface area - used as an approximation of excitatory synapse distributions - as a function of current transfer, and (B, D, F, H) current transfer as a function of path distance from the soma.
Analyses were performed on normalized scales (left panels), where current transfer and path distance are expressed as percentages of their respective maxima, and on absolute scales (right panels). Apical (upper panels) and basal (lower panels) dendrites were analysed separately. Overall differences between UT neurons with activated and deactivated channels were assessed using two-way ANOVA; significant differences are indicated by p values, non-significant differences correspond to *p > 0.05,* are not shown.
Asterisks mark distance ranges or transfer intervals with significant differences between UT neurons with activated and deactivated channels (Bonferroni *post hoc* test, *p < 0.05*).
Distance regions and transfer intervals with fewer than three data points in either group were excluded from statistical analyses.

**Supplementary Figure 2.** Contributions of subthreshold active conductances to steady-state voltage transfer in ultrasound treated CA1 pyramidal neurons with HCN and K_A_ channels active (UT_ON) versus deactivated (UT_OFF).
Comparison graphs illustrate alterations in steady-state voltage transfers of locally generated PSPs in UT neurons with activated HCN and K_A_ channels (orange) relative to UT neurons with deactivated channels (red). Overall differences between UT neurons with activated and deactivated channels were assessed using two-way ANOVA, significant differences are indicated by p values, whereas non-significant differences correspond to *p > 0.05,* are not shown.
Asterisks mark distance ranges or transfer intervals with significant differences between UT neurons with activated and deactivated channels (Bonferroni *post hoc* test, *p < 0.05*).
Distance regions and transfer intervals containing fewer than three data points in either group were excluded from statistical analyses.

**Supplementary Figure 3.** Contributions of subthreshold active conductances to sinusoidal voltage transfer in ultrasound treated CA1 pyramidal neurons with HCN and K_A_ channels active (UT_ON) versus deactivated (UT_OFF).
Comparison graphs illustrate alterations in sinusoidal voltage transfers of locally generated PSPs in UT neurons with activated HCN and K_A_ channels (orange) relative to UT neurons with deactivated channels (red). Overall differences between UT neurons with activated and deactivated channels were assessed using two-way ANOVA, significant differences are indicated by p values, whereas non-significant differences correspond to *p > 0.05,* are not shown.
Asterisks mark distance ranges or transfer intervals with significant differences between UT neurons with activated and deactivated channels (Bonferroni *post hoc* test, *p < 0.05*).
Distance regions and transfer intervals containing fewer than three data points in either group were excluded from statistical analyses.

**Supplementary Figure 4.** Contributions of subthreshold active conductances to propagation delay in ultrasound treated CA1 pyramidal neurons with HCN and K_A_ channels active (UT_ON) versus deactivated (UT_OFF).
Comparison graphs illustrate alterations of propagation delays in UT neurons with activated HCN and K_A_ channels (orange) relative to UT neurons with deactivated channels (red). Overall differences between UT neurons with activated and deactivated channels were assessed using two-way ANOVA, significant differences are indicated by p values, whereas non-significant differences correspond to *p > 0.05,* are not shown.
Asterisks mark distance ranges or delay intervals with significant differences between UT neurons with activated and deactivated channels (Bonferroni *post hoc* test, *p < 0.05*).
Distance regions and propagation delay intervals containing fewer than three data points in either group were excluded from statistical analyses.

**Supplementary note**

I. **Analytical derivation of ultrasound-induced signalling changes using idealized ball-and-stick and multi-dendrite models**

To provide an analytical framework for our 3D numerical simulations and isolate the individual biophysical contributions of ultrasound-induced morphological alterations, we applied classical linear cable theory (Rall, 1959; Jack et al., 1975; Tuckwell, 1988; Major et al., 1993) to idealized compartmental geometries. We first establish a single-dendrite ("ball-and-stick") framework and subsequently expand it to a two-dendrite model to capture the electrical interactions between the apical and basal arbours (Coombes et al., 2007; Timofeeva et al., 2008).

**1. Single-dendrite (ball-and-stick) framework**

Consider an idealized neuron consisting of a somatic sphere (the "ball") with total somatic input conductance

$$G_{S}=\frac{1}{R_{S}}$$

attached to a single unbranched cylindrical dendrite (the "stick") of physical length $l$ and diameter $d$. The characteristic conductance of a semi-infinite cable of identical diameter is defined as:

$$G_{\infty}=\frac{\pi d^{3/2}}{2\sqrt{R_{m}R_{a}}}$$

where $R_{m}$ represents specific membrane resistance and $R_{a}$ represents axial resistivity (Jack et al., 1975). We define the dimensionless electrotonic distance from the soma as $X=\frac{x}{\lambda}$, where $\lambda=\sqrt{\frac{d\cdot R_{m}}{4R_{a}}}$ is the electrotonic length constant, and $L=\frac{l}{\lambda}$ represents the total electrotonic length of the cylinder.

For a steady-state subthreshold current injected at dendritic location $X$ (where $X=0$ defines the soma), assuming the distal dendritic tip ($X=L$) terminates in a sealed end, the steady-state voltage transfer ratio ($A_{V}$) and the current transfer ratio from the injection site to the soma are expressed analytically as (Rall, 1959; Jack et al., 1975; Major et al., 1993):

$$A_{V}\left( X \right)=\frac{V_{soma}}{V\left( X \right)}=\frac{1}{\cosh\left( X \right)+\left( \frac{G_{S}}{G_{\infty}} \right)\sinh\left( X \right)}$$

$$A_{I}\left( X \right)=\frac{I_{soma}}{I_{in}\left( X \right)}=\frac{1}{\cosh\left( X \right)+\left( \frac{G_{\infty}}{G_{S}} \right)\sinh\left( X \right)}$$

This baseline framework reveals two opposing consequences of somatic shrinkage. In ultrasound-treated (UT) neurons, somatic surface area decreases, which, assuming unchanged specific membrane conductance, directly reduces $G_{S}$. Mathematically, a smaller $G_{S}$ decreases the ratio $\left( \frac{G_{S}}{G_{\infty}} \right)$, thereby increasing voltage transfer, while simultaneously increasing the inverse ratio $\left( \frac{G_{\infty}}{G_{S}} \right),$ which reduces current transfer. Furthermore, upward physical segment elongation $\left( l\uparrow\right)$ increases the electrotonic distance $X$, thereby increasing the denominator of the transfer functions and producing bigger spatial attenuation.

**2. Expansion to two idealized dendrites (apical and basal arbours)**

To model the distinct morphological fates of the apical and basal arbours in CA1 pyramidal neurons, we expand the geometry to two idealized cylindrical dendrites (Dendrite 1: Apical; Dendrite 2: Basal) attached to the same somatic sink $G_{S}$ (Coombes et al., 2007; Timofeeva et al., 2008).

When a signal propagates from Dendrite 1 (Apical) toward the soma, the electrical load encountered at the boundary is the sum of the native somatic conductance $G_{S}$ and the input conductance of the attached sister cable, Dendrite 2 $\left( G_{in,2} \right)$. For a dendrite of total electrotonic length $L_{2}$, its somatic input conductance is $G_{in,2}=G_{\infty,2}\tanh\left( L_{2} \right)$ (Rall, 1959). Thus, the effective somatic boundary conductance ($G_{S,eff}$) seen by Dendrite 1 becomes:

$$G_{S,eff}=G_{S}+G_{in,2}=G_{S}+G_{\infty,2}\tanh\left( L_{2} \right)$$

Substituting this effective boundary into our transfer equations yields the analytical multi-dendrite transfer ratios for an apical signal:

$$A_{V,1}\left( X_{1} \right)=\frac{1}{\cosh\left( X_{1} \right)+\left( \frac{G_{S,eff}}{G_{\infty,1}} \right)\sinh\left( X_{1} \right)}$$

$$A_{I,1}\left( X_{1} \right)=\frac{1}{\cosh\left( X_{1} \right)+\left( \frac{G_{\infty,1}}{G_{S,eff}} \right)\sinh\left( X_{1} \right)}$$

**3. Resolving multi-parametric ultrasound alterations**

This multi-dendrite expression captures how the distinct morphological shifts observed in UT neurons interact to govern whole-cell signaling:

**Basal increased spine formation (the inter-arbour shunt):** The basal arbour (Dendrite 2) undergoes spinogenesis, increasing its surface area. Within the equivalent-cylinder description the increase in membrane surface area is represented as an increase in the characteristic conductance ($G_{\infty,2} \uparrow$), which shortens the effective length constant ($\lambda_{2}\downarrow⇰ L_{2}\uparrow). Con$sequently, basal input conductance ($G_{in,2} \uparrow$) rises. This basal increase in surface area acts as an enhanced electrical load ($G_{S,eff}$) at the soma, increasing the shunting of depolarizing voltage arriving from the apical tree and more than compensating for the increase in voltage transfer (**Figure 5D**) produced by somatic shrinkage ($G_{S}\downarrow$).

**Compensating effects governing apical current transfer:** Our NEURON simulations revealed that proximal apical current transfer remained surprisingly unchanged (**Figure 4D**). The analytical multi-dendrite model provides an explanation for this balance: while the reduced somatic conductance decreases current transfer, extensive apical spine pruning increases the effective transmembrane membrane resistance ($R_{m}$ ↑) experienced by the equivalent cable. Because $G_{\infty,1}$ is inversely proportional to $\sqrt{R_{m}}$, apical pruning mathematically decreases $G_{\infty,1}$. This decreases the ratio $\left( \frac{G_{\infty,1}}{G_{S,eff}} \right)$, thereby promoting apical current transfer and compensating for the reduction in transfer associated with the weaker somatic conductance.

**The local voltage rise (**$V_{dend}\uparrow$**):** In the apical arbour, the increase in effective $R_{m}$ increases local input impedance. This increases the local dendritic voltage response ($V\left( X_{1} \right)$) under fixed current injection. Because voltage transfer is the ratio of $V_{soma}$ to local $V\left( X_{1} \right)$, this local voltage elevation tends to drive the overall apical voltage transfer efficiency down.

**Segment elongation dominance (**$l \uparrow⇰ X \uparrow$**):** In basal arbours, widespread segment elongation ($l \uparrow$) increases dimensionless distance $X$. Within the denominators, $\cosh\left( X \right)$ and $\sinh\left( X \right)$increase approximately exponentially with distance for large electrotonic distances ($\cosh\left( X \right), \sinh\left( X \right)\approx\frac{1}{2}e^{X}$ for $X» 1$), causing the effect of increasing electrotonic distance to dominate the reduction in the somatic boundary term and resulting in exponential spatial attenuation over extended paths (**Figure 4H**).

**4. Sinusoidal (AC) voltage transfer**

To evaluate 50 Hz sinusoidal transfers, we apply classical linear AC cable theory (Jack et al., 1975; Tuckwell, 1988). When an alternating voltage of angular frequency $\omega$ propagates along a cylindrical dendrite, its spatial attenuation and phase shift are governed by the complex propagation coefficient $\gamma\left( \omega\right)$, which is obtained by replacing the steady-state membrane resistance with the corresponding frequency-dependent membrane impedance in the cable equation:

$$\gamma\left( \omega\right)=\alpha\left( \omega\right)+j\beta\left( \omega\right)=\frac{1}{\lambda}\sqrt{1+j\omega\tau_{m}}$$

where $\tau_{m}=R_{m}C_{m}$ represents the local membrane time constant, $\alpha\left( \omega\right)$ represents the real attenuation coefficient per unit length (spatial decay), and $\beta\left( \omega\right)$ represents the phase constant.

To determine the physical rate of spatial attenuation without relying on polar trigonometric forms, we square both sides of the propagation equation to separate the real and imaginary components:

$$\left( \alpha^{2}-\beta^{2} \right)+j\left( 2\alpha\beta\right)=\frac{1}{\lambda^{2}}\left( 1+j\omega\tau_{m} \right)$$

Equating the real and imaginary parts of both sides and solving the resulting algebraic system yields the spatial attenuation coefficient per unit length:

$$\alpha\left( \omega\right)=\frac{1}{\lambda}\sqrt{\frac{\sqrt{1+(\omega\tau_{m})^{2}}+1}{2}}$$

In classical cable theory, just as the steady-state length constant $\lambda$ dictates the distance over which a direct current (DC) signal decays by $1/e$, the effective AC length constant ($\lambda_{AC}$) is defined as the inverse of this real attenuation coefficient ($\lambda_{AC}=\frac{1}{\alpha}$). Taking the reciprocal of $\alpha\left( \omega\right)$ provides the explicit, real-valued expression for AC spatial decay:

$$\lambda_{AC}\left( \omega\right)=\lambda\sqrt{\frac{2}{\sqrt{1+(\omega\tau_{m})^{2}}+1}}$$

This formulation provides a direct analytical link between capacitive loading and frequency-dependent signal filtering:

**High-frequency filtering**: Because the denominator of the $\lambda_{AC}$ equation exceeds unity for any nonzero frequency, $\lambda_{AC}$ is shorter than the steady-state length constant $\lambda$. Consequently, AC signals undergo bigger spatial attenuation than steady-state signals along the dendritic arbour (**Figure 5D, H vs. Figure 6D, H**).

**Basal spinogenesis and compensated AC transfer**: In UT neurons, basal spinogenesis adds substantial capacitive loading ($C_{m}\uparrow$). This increases the local membrane time constant ${(\tau}_{m}\uparrow$), which decreases the local AC length constant ($\lambda_{AC}$ $\downarrow$), thereby strengthening low-pass filtering along the basal dendrites relative to the apical dendrites, where spine loss reduces the effective membrane capacitance and membrane time constant (**Figure 6D vs. Figure 6H**). However, our simulations demonstrate that the overall basal sinusoidal voltage transfer to the soma remains unaltered (**Figure 6H**) after US treatment. This stability occurs because the increased spatial attenuation along the basal path is largely offset by the concurrent reduction in the somatic sink ($G_{S}\downarrow$). The reduced somatic conductance requires less AC current to generate a given somatic voltage response, thereby compensating for the bigger dendritic attenuation and preserving a similar somatic voltage amplitude. These opposing effects provide a mechanistic explanation for the preservation of basal 50 Hz voltage transfer observed in the simulations.

**5. Somatopetal propagation delay**

Propagation delay across branching geometries is analytically quantified by the difference between the centers of gravity (centroids) of the voltage waveforms (Agmon-Snir & Segev, 1993). For an idealized cable of physical length $x$, local centroid propagation delay scales quadratically:

$$\tau_{cent}\left( x \right)\approx\frac{r_{a}c_{m}}{2}x^{2}+r_{a}c_{m}x\left( \frac{G_{sink}}{G_{\infty}} \right)$$

where $r_{a}$ and $c_{m}$ represent axial resistance and membrane capacitance per unit length, respectively.

**Apical spine pruning acceleration** ${(c}_{m}\downarrow$) Our simulations revealed a statistically significant decrease in propagation delays (faster signalling) within the apical arbour on the absolute scale (**Figure 7D**). The analytical model confirms this: massive spine pruning effectively removes parallel membrane surface area, reducing the specific capacitance per unit length ${(c}_{m}\downarrow$). This decreases the capacitive load in the delay equation, forcing the signal to propagate faster toward the soma.

**The quadratic dependence on path length (**$x^{2}$**):** Centroid delay scales with the square of physical path distance ($x^{2}$). Because ultrasound exposure induces segment elongation in the basal arbour ($x \uparrow$), propagation delay increases quadratically over longer paths (**Figure 7H**).

**Capacitive loading from basal branching and spines (**$c_{m}\uparrow$**):** In the basal arbour, spinogenesis increases the capacitance per unit length ($c_{m}$). Because additional bifurcations and spines increase the membrane capacitance per unit length, more charge is required to change the membrane potential of the expanded membrane surface. This increases$c_{m}$ in the analytical expression, providing a mechanistic explanation for the prolonged somatopetal propagation delays observed in the basal dendrites of UT neurons (**Figure 7H**).

These analytical expressions are not intended to reproduce the full complexity of the reconstructed neuronal morphologies. Rather, they isolate the principal cable-theoretic consequences of the observed morphological alterations and provide a mechanistic framework for interpreting the corresponding NEURON simulation results.

**II. Quantitative illustration of the analytical predictions using the ball-and-stick model**

To illustrate the predictive capacity of the analytical expressions, we inserted representative morphometric values measured from the reconstructed neurons into the ball-and-stick equations. These calculations are intended as order-of-magnitude estimates rather than exact quantitative predictions because the analytical model neglects dendritic tapering, branch asymmetry, and other anatomical complexities incorporated into the full NEURON simulations. Nevertheless, they demonstrate that the analytical framework predicts both the direction and approximate magnitude of the observed signalling changes.

**1. Approximate balance governing apical current transfer**

Morphometric analysis showed that the mean somatic surface area decreased from 218 μm² to 140 μm² in UT neurons, corresponding to approximately 64% of the control value. Assuming unchanged specific membrane conductance, the somatic conductance therefore scales proportionally,

$G_{S,UT}\approx0.64 G_{S,NT}$.

If this were the only morphological alteration, the ratio $\left( \frac{G_{\infty}}{G_{S}} \right)$ appearing in the current-transfer equation would increase by a factor of approximately 1/0.64=1.56, predicting a substantial reduction in current transfer efficiency.

Apical spine pruning acts in the opposite direction. Because CA1 apical dendrites possess a large spine membrane contribution, the loss of spines increases the effective transmembrane resistance of the equivalent cable. Incorporating the measured reductions in spine density and individual spine surface area into the cable equations yields approximately a two-fold increase in effective transverse membrane resistance. Since

$G_{\infty}\propto\frac{1}{\sqrt{R_{m,\text{eff}}}}$ ,

the characteristic conductance decreases to approximately $\frac{1}{\sqrt{2.0}}\approx0.71$ of its control value.

When these two morphological factors interact in the denominator of the current transfer equation, the new ratio becomes

$\left( \frac{0.71 G_{\infty,NT}}{0.64 G_{S,NT}} \approx1.11 \right)$,

indicating that the large effect expected from somatic shrinkage alone is substantially reduced once apical spine pruning is taken into account. Moreover, because this ratio multiplies the hyperbolic sine term, which remains small for proximal electrotonic distances, the resulting effect on the total denominator of the transfer equation is modest. Thus, the analytical model predicts only a small alteration in proximal apical current transfer, consistent with the NEURON simulations (**Figure 4D**).

**2. Approximation of asymmetric delays**

The centroid delay equation predicts

$\tau_{cent}\left( x \right)\propto x^{2}c_{m}$ ,

indicating that propagation delay depends on both path length and membrane capacitance.

For the basal arbor, morphometric analysis demonstrated both segment elongation and increased spine membrane area. Morphological data extracted from the reconstructed neurons indicate that modest increases in path length and effective membrane capacitance combine multiplicatively in the analytical expression, producing an estimated delay increase of approximately 50%. This closely matches the direction and order of magnitude of the basal slowing observed in the NEURON simulations (**Figure 7H**).

For the apical arbor, segment lengths remained largely unchanged, whereas spine pruning reduced the effective membrane capacitance. Consequently, the analytical model predicts a proportional reduction in delay, consistent with the faster apical signalling observed in the simulations (**Figure 7D**).

These calculations are intended to demonstrate that the simplified cable-theory equations capture the dominant trends produced by the measured morphological alterations, rather than to reproduce the precise quantitative behaviour of the full three-dimensional neuronal reconstructions.

**References**

Agmon-Snir, H., & Segev, I. (1993). Signal delay and input synchronization in passive dendritic structures. *Journal of Neurophysiology*, 70(5), 2066–2085.

Coombes, S., Timofeeva, Y., Svensson, C. M., Lord, G. J., Josić, K., Cox, S. J., & Gregory, M. D. (2007). Branching dendrites with resonant membrane: a "sum-over-trips" approach. *Biological Cybernetics*, 97(2), 137–149.

Jack, J. J. B., Noble, D., & Tsien, R. W. (1975). *Electric current flow in excitable cells*. Oxford: Clarendon Press.

Major, G., Evans, J. J., & Jack, J. J. B. (1993). Solutions for transients in arbitrarily branching cables: I. Voltage recording with a somatic shunt. *Biophysical Journal*, 65(1), 423–449.

Rall, W. (1959). Branching dendritic trees and motoneuron membrane resistivity. *Experimental Neurology*, 1(5), 491–527.

Timofeeva, Y., Cox, S. J., Coombes, S., & Josić, K. (2008). Democratization in a passive dendritic tree: an analytical investigation. *Journal of Computational Neuroscience*, 25(2), 228–244.

Tuckwell, H. C. (1988). *Introduction to theoretical neurobiology: Volume 1, Linear cable theory and dendritic structure*. Cambridge University Press.
